# Risk Variants in the Exomes of Children With Critical Illness

**DOI:** 10.1001/jamanetworkopen.2022.39122

**Published:** 2022-10-28

**Authors:** Joshua E. Motelow, Natalie C. Lippa, Joseph Hostyk, Evin Feldman, Matthew Nelligan, Zhong Ren, Anna Alkelai, Joshua D. Milner, Ali G. Gharavi, Yingying Tang, David B. Goldstein, Steven G. Kernie

**Affiliations:** 1 Institute for Genomic Medicine, Columbia University Medical Center, New York, New York; 2 Division of Critical Care and Hospital Medicine, Department of Pediatrics, Columbia University Irving Medical Center, NewYork-Presbyterian Morgan Stanley Children's Hospital, New York, New York; 3 Regeneron Genetics Center, Regeneron Pharmaceuticals, Tarrytown, New York; 4 Division of Nephrology, Department of Medicine, Columbia University Irving Medical Center, NewYork-Presbyterian, New York, New York; 5 Molecular Genetics Laboratory, New York City Office of Chief Medical Examiner, New York, New York; 6 NewYork-Presbyterian Hospital, New York, New York

## Abstract

**Importance:**

Diagnostic genetic testing can lead to changes in management in the pediatric intensive care unit. Genetic risk in children with critical illness but nondiagnostic exome sequencing (ES) has not been explored.

**Objective:**

To assess the association between loss-of-function (LOF) variants and pediatric critical illness.

**Design, Setting, and Participants:**

This genetic association study examined ES first screened for causative variants among 267 children at the Morgan Stanley Children’s Hospital of NewYork-Presbyterian, of whom 22 were otherwise healthy with viral respiratory failure; 18 deceased children with bronchiolitis from the Office of the Chief Medical Examiner of New York City, of whom 14 were previously healthy; and 9990 controls from the Institute for Genomic Medicine at Columbia University Irving Medical Center. The ES data were generated between January 1, 2015, and December 31, 2020, and analyzed between January 1, 2017, and September 2, 2022.

**Exposure:**

Critical illness.

**Main Outcomes and Measures:**

Odds ratios and *P* values for genes and gene-sets enriched for rare LOF variants and the loss-of-function observed/expected upper bound fraction (LOEUF) score at which cases have a significant enrichment.

**Results:**

This study included 285 children with critical illness (median [range] age, 4.1 [0-18.9] years; 148 [52%] male) and 9990 controls. A total of 228 children (80%) did not receive a genetic diagnosis. After quality control (QC), 231 children harbored excess rare LOF variants in genes with a LOEUF score of 0.680 or less (intolerant genes) (*P* = 1.0 × 10^−5^). After QC, 176 children without a diagnosis harbored excess ultrarare LOF variants in intolerant genes but only in those without a known disease association (odds ratio, 1.8; 95% CI, 1.3-2.5). After QC, 25 children with viral respiratory failure harbored excess ultrarare LOF variants in intolerant genes but only in those without a known disease association (odds ratio, 2.8; 95% CI, 1.1-6.6). A total of 114 undiagnosed children were enriched for de novo LOF variants in genes without a known disease association (observed, 14; expected, 6.8; enrichment, 2.05).

**Conclusions and Relevance:**

In this genetic association study, excess LOF variants were observed among critically ill children despite nondiagnostic ES. Variants lay in genes without a known disease association, suggesting future investigation may connect phenotypes to causative genes.

## Key Points

**Question:**

Do the exomes of critically ill children without a genetic diagnosis carry risk factors?

**Findings:**

This genetic association study of 267 children with critical illness and 18 deceased children found a statistically significant burden of predicted loss-of-function variants in cases compared with 9990 controls among genes intolerant to loss-of-function variation.

**Meaning:**

Children with critical illness may carry loss-of-function risk variants for their clinical presentation regardless of whether a causative diagnostic variant is identified.

## Teaser

This genetic association study of deceased children and children with critical illness examines whether loss-of-function variants exist among critically ill children with nondiagnostic exome sequencing.

## Introduction

Children with monogenic or chromosomal abnormalities account for 16% of pediatric admissions and have increased mortality risk in pediatric intensive care units (PICUs).^1,2,3,4^ Whole exome sequencing (ES) and genome sequencing (GS) achieve diagnoses in 24% to 52% of children with critical illness and can lead to changes in management.^5,6,7,8,9,10,11,12,13^ Among patients with nondiagnostic ES (ndES), an explanatory variant may be unreported because it was (1) undetected by ES, (2) detected by ES but not determined to be causative, or (3) detected by ES in a gene of unknown significance, among other reasons.^14,15^

Bronchiolitis is a viral infection of the lower respiratory tract.^16^ Nearly every child will be exposed to a virus that causes bronchiolitis.^17^ Of children admitted to the hospital for bronchiolitis, approximately 9% require ventilatory support, and death occurs in less than 0.1%.^18,19^ Although risk factors for hospitalization have been identified, including prematurity, most hospitalized children are otherwise healthy.^16,19,20,21^ Genetic risk factors have been proposed, but a definitive answer is elusive.^22,23,24,25,26^ We hypothesized that children with critical illness and ndES carry deleterious variants in genes without a known disease association. These variants are risk factors for the child’s illness but do not yield a diagnosis because the genes are not currently associated with disease. If such an association existed, larger association studies could uncover causative genes for pediatric critical illness.

## Methods

### Study Population

Children with critical illness were enrolled through 2 arms: (1) critically ill children at New York-Presbyterian Morgan Stanley Children’s Hospital/Columbia University Irving Medical Center (MSCH/CUIMC; MSCH cohort, n = 267) and (2) deceased children from the Office of the Chief Medical Examiner (OCME) of New York City (OCME cohort, n = 18). Critical illness in the MSCH cohort was defined by admission to the PICU or respiratory failure from a virus requiring noninvasive positive pressure. The OCME data were collected from January 1, 2003, to December 31, 2016, using a keyword search for *viral bronchiolitis* in the immediate cause of death. Of 24 cases identified, 18 had adequate samples for ES (Table 1; eTable 1 in the Supplement). We defined a combined cohort by merging the MSCH and OCME cohorts (n = 285) (Table 1). A viral respiratory failure cohort was created by combining healthy probands from the MSCH cohort with respiratory failure secondary to a virus (n = 22) (eTable 2 in the Supplement) and (2) probands from the OCME cohort with no phenotype data suggestive of chronic illness or an alternative cause before death (n = 14) (eTable 1 in the Supplement). Children admitted for trauma or who were 19 years or older were excluded. Controls were individuals with data at the Institute for Genomic Medicine (IGM) enrolled as controls (n = 3358) or healthy family members (n = 6632) across multiple studies. The Columbia University Institutional Review Board approved protocols, and written informed consent was provided for using DNA in genetic research at the IGM at CUIMC.^27^ Individuals enrolled at the MSCH were referred by their CUIMC clinician. Written informed consent was provided by the legal guardian for participants younger than 18 years or by the individual when 18 years or older. This report follows the guidelines for case-control genetic association studies as outlined by the Strengthening the Reporting of Genetic Association Studies (STREGA) reporting guideline.

**Table 1.** Demographic Characteristics of 285 Children With Critical Illness

Characteristic	No. (%)	Age, median (IQR) [range], y
Total cohort	285 (100)	4.1 (0.9-11.6) [0.0-18.9]
Sex		
Female	137 (48)	4.8 (1.0-12.7) [0.0-18.9]
Male	148 (52)	3.4 (0.9-10.6) [0.1-18.4]
Enrollment location		
Clinic	131 (46)	5.2 (2.1-12.4) [0.0-18.6]
PICU^a^	136 (48)	3.6 (0.7-11.9) [0.0-18.9]
OCME	18 (6)	0.3 (0.1-0.9) [0.0-1.8]
Diagnostic status		
Resolved	46 (16)	4.3 (0.7-11.5) [0.1-18.0]
Partially resolved	11 (4)	5.1 (2.2-9.0) [0.2-15.3]
Unresolved	228 (80)	3.7 (1.0-11.7) [0.0-18.9]
Viral respiratory failure cohort		
MSCH	22 (61)	1.0 (0.2-1.7) [0.0-9.6]
OCME	14 (39)	0.2 (0.1-0.4) [0.0-1.8]
Geographic ancestry		
African	87 (31)	NR
East Asian	12 (4)	NR
European	42 (15)	NR
Latino	92 (32)	NR
Middle Eastern	1 (0)	NR
South Asian	14 (5)	NR
Admixed	37 (13)	NR
Trios		
Resolved	46	4.1 (0.7-10.0) [0.1-17.6]
Unresolved	114	3.6 (1.0-9.9) [0.0-18.9]

Abbreviations: MSCH, NewYork-Presbyterian Morgan Stanley Children’s Hospital; NR, not reported; OCME, Office of the Chief Medical Examiner; PICU, pediatric intensive care unit.

^a^ Includes 1 proband with a PICU admission at an outside hospital and 2 children enrolled from the general pediatric ward but still meeting criteria for respiratory failure based on respiratory failure that required noninvasive positive pressure ventilation (eMethods in the Supplement).

### Next-Generation Sequencing, Variant Calling, and Quality Control

Sequencing was performed at or transferred to the IGM. Case data are from ES. Controls underwent ES (n = 9353) or GS (n = 637). Established protocols have been used at the IGM to generate ES or GS for more than 120 000 individuals (eMethods in the Supplement).^28,29,30^ Variants were annotated with an in-house analysis tool for annotated variants (ATAV) platform.^31^

### Diagnostic Analysis

The diagnostic analysis of the MSCH cohort is a subset of that reported by Lippa et al.^27^ Briefly, trio (ie, ES available from proband and both biological parents) or proband-only ES analyses were completed. Candidate genotypes were reviewed with the research and clinical teams to reach consensus. Variants deemed causative were confirmed in a Clinical Laboratory Improvement Amendments–certified laboratory before return to patients and clinicians. Only single-nucleotide variants and insertion/deletions were analyzed. No other variants were assessed in the research pipeline. Individuals may have also undergone genetic evaluation by their clinical team.

Probands harboring genetic findings that explained all or part of their phenotypes were deemed resolved (n = 57) (Table 1). Genetic findings were drawn from the research pipeline and medical record review. Six of the 57 probands were resolved outside this research pipeline. The remainder were considered the unresolved cohort (n = 228). The ES from these probands were considered nondiagnostic ES (ndES). Participants recruited from the OCME underwent a modified diagnostic pipeline because of limited phenotype information (eTable 1 in the Supplement). No explanatory variants were identified, and all were considered unresolved.

### Statistical Analysis

#### Clustering

We matched cases and controls by geographic ancestry using clustering as previously described because it is important to correct for the underlying rate of variation in samples of different geographic ancestry in case-control experimental designs (eFigure 1 in the Supplement).^32,33,34^ Principal component analysis for dimensionality reduction was performed on a set of predefined variants to capture population structure.^35^ Clusters were identified using the Louvain method of community detection using the first 6 principal components as input, which reflected geographic ancestry.^32,36^

#### Gene-Based Collapsing

Collapsing refers to an analysis in which variants satisfying specific criteria (qualifying variants [QVs]) are considered equivalent and a statistical association is determined between harboring a QV in a gene or gene set and case-control status. The QV specification determines the collapsing model (eTable 3 in the Supplement).^32,34,37,38^ For each gene and individual within each cluster, an indicator variable (1/0 states) was assigned if a QV in the gene was present (state 1) or not (state 0) to create a gene × individual matrix for each cluster. From the collapsing matrixes of each cluster, we extracted the number of cases and controls with and without a QV per gene and tested for an enrichment in cases or controls.^32,34,39,40,41^

#### LOF Analysis

**Figure 1.**
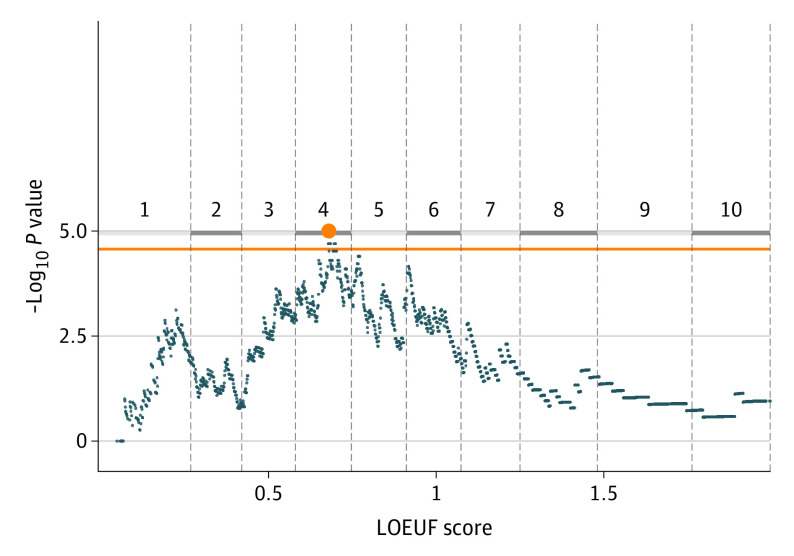
Loss-of-Function (LOF) Variants in Children With Critical Illness We assessed excess LOF variants in our combined case-control cohort (231 cases and 5322 controls) in gene sets defined by loss-of-function observed/expected upper bound fraction (LOEUF) scores. Gene sets to the left of the plot are more intolerant to LOF variation compared with subsets of gene sets to the right of the plot. For each gene set, enrichment was evaluated and a *P* value was obtained. Empirical *P* values (y-axis) were determined by 100 000 permutations. The horizontal orange line represents significance using Bonferroni correction. The minimum empirically derived *P* value, which indicates the most significant enrichment across all LOEUF thresholds, occurs at a LOEUF of 0.680 or less (orange dot). The 1 to 10 labels indicate the intolerance decile based on LOEUF scores, with 1 representing the 10% most intolerant genes and 10 representing the 10% least intolerant genes.

We assessed the optimal gene intolerance threshold associating case-control status and QV carrier status (Figure 1).^15^ In the rare loss-of-function (LOF) model (eTable 3 in the Supplement) in the combined cohort, 8702 genes with 1860 unique loss-of-function observed/expected upper bound fraction (LOEUF) scores harbored QVs.^42^ Genes were ordered based on LOEUF scores from most to least intolerant to LOF variation, and gene sets were created for genes less than or equal to each LOEUF threshold. For each gene set, we calculated a *P* value using the Cochran-Mantel-Haenszel test that signified the statistical association of case-control status and LOF carrier status. Empirical *P* values were determined by 100 000 permutations of the Cochran-Mantel-Haenszel *P* values. We used a Bonferroni correction for the 1860 comparisons to assess significance. A 2-sided *P* < 2.7 × 10^−5^ was considered to be statistically significant.

#### Gene Set Collapsing

We tested associations of case-control status with QV status in these gene sets. Genes with a confirmed phenotype in Online Mendelian Inheritance in Man (4145 genes), Development Disorder Genotype-Phenotype Database (877 genes), and The Human Gene module of SFARI Gene (1898 genes) were considered to be associated with a disease.^43,44,45,46^ Combining these yielded 4662 unique disease-associated genes (eMethods in the Supplement). Of these, 2274 harbored a variant in a nonsynonymous model. Of the genes without a known association (n = 13 624), 6478 harbored a variant in a nonsynonymous model. To assess potential associations among the viral respiratory failure cohort, we included gene lists for primary immunodeficiencies, primary ciliary dyskinesia, asthma, and genes associated with susceptibility to life-threatening viral illness (eMethods and eTable 4 in the Supplement).^47^ We used a false discovery rate–adjusted *P* value for multiple comparisons. We performed 22 Cochran-Mantel-Haenszel or Fisher exact tests to determine odds ratios for gene set enrichment testing and defined a significant enrichment at a false discovery rate–adjusted *P* < .05.

#### De Novo Mutation Calling, Filtering, and Analysis

We obtained de novo variants for probands with both biological parents enrolled. To test the null hypothesis that our cohort contained an expected number of de novo variants in genes without a known disease association, we used denovolyzeR, version 0.2.0.^48,49,50,51^ denovolyzeR is an R package that compares the rate of de novo variants in a data set to an expected rate to assess enrichment.

## Results

### Diagnostic Outcomes and Phenotypes

This analysis included 285 children (148 [52%] male and 137 [48%] female; 87 [31%] African, 12 [4%] East Asian, 42 [15%] European, 92 [32%] Latino, 1 [0%] Middle Eastern, 14 [5%] South Asian, and 37 [13%] admixed geographic ancestry) with critical illness (Table 1) enrolled from the MSCH/CUIMC or OCME who were 18 years or younger at enrollment (median [range] age, 4.1 [0-18.9] years). Fifty-seven of 285 (20%) achieved a full or partial diagnosis (eTable 5 in the Supplement). Enrolled phenotypes were heterogenous (eMethods and eTable 6 in the Supplement) but typical of PICU admissions.^18,52,53,54^

### Clustering and Collapsing

A total of 231 cases and 5322 controls in the combined cohort, 176 cases and 5180 controls in the unresolved cohort, and 25 cases and 2973 controls in the viral respiratory failure cohort were analyzed after quality control and ancestry matching (eTable 7 in the Supplement) for cluster-based, rare variant collapsing association analyses. For each cohort, 2 models were tested with LOF variants (eTable 3 in the Supplement). The ultrarare LOF model included only variants absent from public data sets, whereas the flex LOF model allowed variants with a minor allele frequency less than 0.1%. No model showed a single gene association with study-wide significance (eFigures 2-10 and eTables 8-16 in the Supplement). The included clusters encompassed samples of European, Latino, and African geographic descent (eFigure 1 in the Supplement). The ultrarare synonymous variant association analysis functioned as a control, and the low genomic-inflation factor indicated adequate ancestry substructure matching (eFigures 2-4 and eTables 8-10 in the Supplement).

### LOF Burden in Children With Critical Illness

We assessed whether children with critical illness carried excess LOF variants and whether this association was mediated by genic intolerance (Figure 1).^15^ We identified 8702 unique genes that harbored a rare LOF variant in either a case or control (minor allele frequency <0.1%, flex LOF model) (eFigure 8 and eTables 3 and 14 in the Supplement), which comprised 1860 unique LOEUF scores.^42^ The LOEUF score quantifies a gene’s intolerance to LOF variation. We created 1860 gene sets composed of genes less than or equal to the unique LOEUF scores and ordered the gene sets from most to least intolerant. We assessed the association of children with critical illness and carrier status in each gene set. The most statistically significant association between case-control status and LOF variants occurred at a LOEUF threshold of 0.680 (*P* = 1.0 × 10^−5^ by permutation).

**Figure 2.**
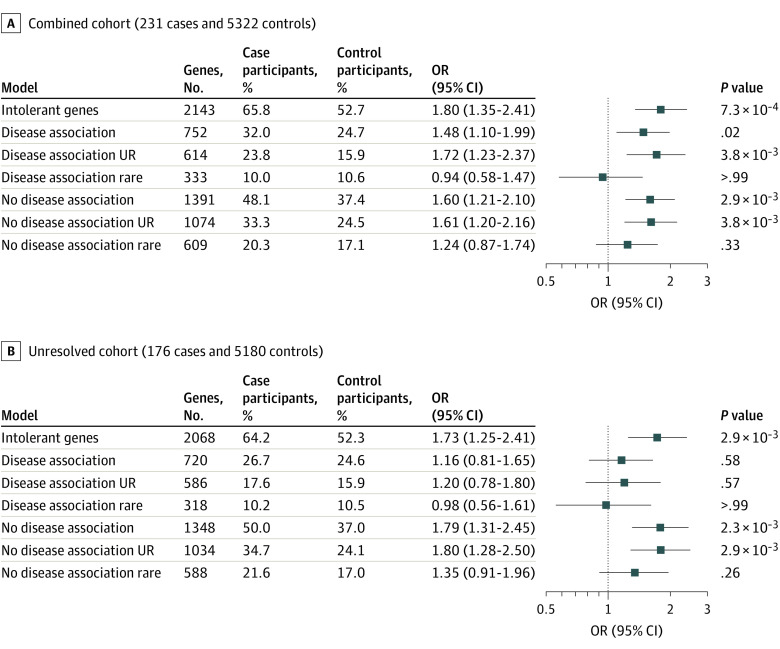
Gene Set Enrichment Analysis Juxtaposing the Combined Cohort Against the Unresolved Cohort Variants were divided into those in genes with a disease (disease association) and genes without a known disease association (no disease association) and ultrarare (UR) (absent from external control data sets) and rare (minor allele frequency <0.1% but present in external control data sets). Only genes with a loss-of-function observed/expected upper bound fraction (LOEUF) score of 0.680 or less (intolerant genes) were included. The genes column indicates the number of genes in the gene set with a qualifying variant in either a case or control participant. Pooled odds ratio (ORs), 95% CIs, and false discovery rate–corrected *P* values were generated from the exact 2-sided Cochran-Mantel-Haenszel test.

We asked 3 questions: Are the variants present in public databases? Are the genes that harbor the variants associated with diseases? Is the association driven by children with a genetic diagnosis? We considered genes with a LOEUF score of 0.680 or less (intolerant genes) (Figure 1). In the combined cohort (Figure 2A; eTable 17 in the Supplement), the association between LOF variants and children with critical illness existed in genes with (OR, 1.5; 95% CI, 1.1-2.0; adjusted *P* = .02) and without (OR, 1.6; 95% CI, 1.1-2.1; adjusted *P* = 2.9 × 10^−3^) known disease associations. The association was significant for variants absent from public data sets (ultrarare) in genes with (OR, 1.7; 95% CI, 1.2-2.4; adjusted *P* = 3.8 × 10^−3^) and without (OR, 1.6; 95% CI, 1.2-2.2; adjusted *P* = 3.8 × 10^−3^) known disease associations. Carrying variants that were rare (minor allele frequency <0.1%) but still present in public data sets was not associated with case-control status.

We removed children who carried variants that partially or fully explained their phenotypes to assess probands with ndES (unresolved cohort) (Figure 2B; eTable 18 in the Supplement). The association of carrying an LOF variant in genes with a disease association was no longer significant (OR, 1.2; 95% CI, 0.8-1.6; adjusted *P* = .58). The association in genes without a known disease association remained significant (OR, 1.8; 95% CI, 1.3-2.5; adjusted *P* = 2.3 × 10^−3^). As in the combined cohort, this association extended only to variants absent from public databases (OR, 1.8; 95% CI, 1.3-2.5; adjusted *P* = 2.9 × 10^−3^).

### Association of Viral Respiratory Failure With Genetic Risk

**Figure 3.**
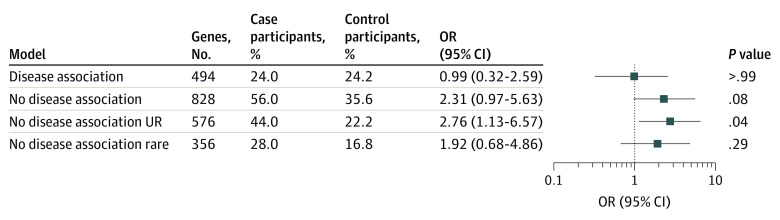
Children With Viral Respiratory Failure Harbor Ultrarare Loss-of-Function Genetic Risk Factors Data include 25 cases compared with 2973 controls. Qualifying variants were divided into those in genes with a disease (disease association) and genes without a known disease association (no disease association) and ultrarare (UR) (absent from external control data sets) and rare (minor allele frequency <0.1% but present in external control data sets). Only genes with a loss-of-function observed/expected upper bound fraction (LOEUF) score of 0.680 or less (intolerant genes) were included. The genes column indicates the number of genes in the gene set with a qualifying variant in either a case or control participant. Odds ratios (ORs), 95% CIs, and false discovery rate–corrected *P* values were generated with the Fisher exact test.

We assessed whether harboring ultrarare LOF variants in intolerant genes was a risk factor for viral respiratory failure (Figure 3; eTable 19 in the Supplement). No association was found between case-control designation and carrier status of LOF variants in genes with a disease association (OR, 1.0; 95% CI, 0.3-2.6; adjusted *P* > .99). For genes without a known disease association, the association with ultrarare variants was significant (OR, 2.8; 95% CI, 1.1-6.6; adjusted *P* = .04), but the association with rare variants present in public databases was not (OR, 1.9; 95% CI, 0.7-4.9; adjusted *P* = .29). No significant association was found among genes implicated in primary immunodeficiencies, asthma, primary ciliary dyskinesia, or prior studies of viral respiratory failure (eTable 20 in the Supplement).^47,55,56,57,58,59,60,61,62^

The viral respiratory failure cohort sample size was inadequate to assess gene-specific associations (eFigures 7 and 10 and eTables 13 and 16 in the Supplement). We highlighted 9 candidate intolerant genes without a known disease association harboring ultrarare LOF variants in cases but not controls (eTable 21 in the Supplement). *CTTN* (OMIM 164765) is implicated in lung disease among patients of African descent,^63^ *RSBN1L* (HGNC 24765) is upregulated in COVID-19,^64^ and *RASGRP4* (OMIM 607320) regulates dendritic and mast cell function.^65,66^

### De Novo Enrichment in Children With Critical Illness

Among the 57 resolved or partially resolved probands, 46 were trios, and among the 227 unresolved probands, 114 were trios (Table 1). We assessed enrichment of de novo variants in genes without a known disease association (Table 2; eTable 8 in the Supplement).^48,50^ De novo LOF variants were enriched in unresolved probands (observed, 14; expected, 6.8; enrichment, 2.05; *P* = .01) but not in resolved probands (observed, 3; expected, 2.8; enrichment, 1.09; *P* = .52). These data support the hypothesis that despite ndES probands in the PICU likely carry LOF risk variants.

**Table 2.** Enrichment of De Novo Variants in Children With Critical Illness in Genes Without a Known Disease Association

Variant class	Observed	Expected	Enrichment^a^	*P *value^b^
**Resolved cohort (n = 46)**				
Synonymous	9	9.30	0.97	.58
Missense	11	20.50	0.54	.99
LOF	3	2.80	1.09	.52
Missense and LOF	14	23.30	0.60	.98
All	23	31.30	0.74	.95
**Unresolved cohort (n = 114)**				
Synonymous	12	23.00	0.52	.99
Missense	47	50.80	0.93	.72
LOF	14	6.80	2.05	.01
Missense and LOF	61	57.60	1.06	.35
All	73	77.50	0.94	.71

Abbreviation: LOF, loss of function.

^a^ Only genes without a known disease association were assessed.

^b^ denovolyzeR was used to assess for de novo enrichment against the predicted null distribution. *P* values were not adjusted for multiple comparisons.

## Discussion

In this study, the first to our knowledge of exomes of critically ill children without a genetic diagnosis, we found that ndES may identify disease-risk variants in genes without a known disease association. The diagnostic rate of our cohort was 20% (Table 1). Carrying LOF variants in genes with a LOEUF score of 0.680 or less (intolerant genes) was significantly associated with critical illness (Figure 1). In the combined cohort, intolerant genes with and without a known disease association harbored excess LOF variants (Figure 2A). Among children without a genetic diagnosis, only associations with genes without a known disease association remained (Figure 2B). Children with viral respiratory failure harbored excess LOF variants in intolerant genes without a known disease association (Figure 3). Among undiagnosed children with critical illness, we found more de novo LOF variants than expected in genes without a known disease association (Table 2).

Our diagnostic rate was lower than that of other PICU cohorts, but our inclusion criteria allowed enrollment whether or not the proband was suspected to have a monogenic condition. Prior studies^5,6,7,8,9,10,11,12,13^ enrolled patients with a high pretest probability of diagnosis. We hypothesized that children with critical illness, regardless of genetic diagnosis, harbored excess LOF variants among genes intolerant to LOF variation but did not know the degree of intolerance. We used an unbiased approach to finding the optimal intolerance threshold.^15^ The most significant association occurred at a LOEUF threshold of 0.680 or less, which approximately represents the most intolerant tertile of genes. A statistically significant threshold identifying genes in the most intolerant tertile of the genome supports the hypothesis that critically ill children are more likely to harbor LOF variants regardless of genetic diagnosis. The LOEUF value in the PICU population can be compared with other cohorts. Stillbirth cases undergoing a similar analysis had a peak threshold of 0.239.^15^ Stillbirth is a more severe phenotype; therefore, we might expect the LOEUF threshold to capture more intolerant genes than when children survived the neonatal period. The connection among intolerance, disease onset, and exposure (eg, viral infection) should be further explored.

Excess LOF variants among critically ill children with ndES in genes without a known disease association implies that these variants are risk factors for their phenotype, but no diagnosis is made because the gene-disease association is not understood.^67^ Comparing the rate of carrier status of ultrarare LOF variants in intolerant genes in the unresolved cohort (34.7% cases vs 24.1% controls) suggests that approximately 10% of these cases will ultimately receive a genetic diagnosis after gene-disease association discovery. Among children with viral respiratory failure, a finding of excess LOF variants (Figure 3) indicates that larger sample sizes could uncover gene-disease associations.^34^ Associations with ultrarare variants support the hypothesis that rare diseases (eg, pediatric critical illness) are driven by variants absent in healthy data sets.^41^

### Limitations

This study has some limitations. Case sample sizes are small, which biases the findings toward spurious associations. The diagnostic assessment was not held to the regulatory standards of a clinical laboratory, included only single-nucleotide variants and insertion/deletions, included additional genetic testing inconsistently, and analyzed the OCME phenotype with restricted phenotype data. Thus, inclusion of potentially resolved probands in the unresolved cohort may have depressed associations in the unresolved cohort. The enrollment in the PICU was not comprehensive, so the diagnostic rate should be interpreted with caution. Given the limited phenotype data, including the OCME probands in the viral respiratory failure cohort, may bias associations toward genes of multisystem disease rather than isolated viral respiratory failure. Early respiratory viral infection is linked to wheezing and asthma.^68,69,70^ Given the challenges of diagnosing asthma in young children, viral respiratory failure associations may be confounded with genes for asthma susceptability.^62^ For the control population, we could not exclude a history of critical illness, which would bias associations toward controls but only marginally given the phenotype’s rarity. The preponderance of probands of African geographic descent from the OCME (eTable 1 in the Supplement) is concerning given high rates of mortality among Black infants.^71^ With limited information available, accounting for nongenetic confounds was challenging. Although generalizability may be questioned because this is a single-center study, the cohort was typical of PICUs (eTable 6 in the Supplement),^18,52^ suggesting the genetic findings are likely found broadly.

## Conclusions

This genetic association study found that critically ill children harbored excess LOF variants. Among children without a genetic diagnosis, these variants were ultrarare and in genes without a known disease association. Associations remained true among a subset with viral respiratory failure despite a small sample size. Among children without a genetic diagnosis, we found excess de novo LOF variants in genes without a known disease association. Findings from this study indicate that critically ill children harbor ultrarare risk variants detected by ES even in the absence of genetic diagnoses.

## Notes

Supplement. **eTable 1.** Probands recruited from the Office of the Chief Medical Examiner of New York City (OCME) **eTable 2.** Summary of MSCH Respiratory Failure Cohort **eTable 3.** Collapsing Model Qualifying Variant Definition **eTable 4.** Genes Associated With Viral Respiratory Failure **eTable 5.** Summary of Diagnostic Variants **eTable 6.** Top Human Phenotype Ontology Terms **eTable 7.** Samples Removed During Data Cleaning **eTable 8.** Top 10 Genes in Ultra-Rare Synonymous Model in Combined Cohort **eTable 9.** Top 10 Genes in Ultra-Rare Synonymous Model in Unresolved Cohort **eTable 10.** Top 10 Genes in Ultra-Rare Synonymous Model in Respiratory Failure Cohort **eTable 11.** Top 10 Genes in Ultra-Rare Loss-of-Function Model in Combined Cohort **eTable 12.** Top 10 Genes in Ultra-Rare Loss-of-Function Model in Unresolved Cohort **eTable 13.** Top 10 Genes in Ultra-Rare Loss-of-Function Model in Respiratory Failure Cohort **eTable 14.** Top 10 Genes in Flex Loss-of-Function Model in Combined Cohort **eTable 15.** Top 10 Genes in Flex Loss-of-Function Model in Unresolved Cohort **eTable 16.** Top 10 Genes in Flex Loss-of-Function Model in Respiratory Failure Cohort **eTable 17.** Data for Gene-Set Enrichment Analysis for Combined Cohort **eTable 18.** Loss-of-Function Burden in Children With Critical Illness but Without a Causative Genetic Diagnosis **eTable 19.** Loss-of-Function Burden in Children With Viral Respiratory Failure in Genes With and Without a Known Disease Association **eTable 20.** Loss-of-Function Burden in Children With Viral Respiratory Failure in Immunodeficiency and Primary Ciliary Dyskinesia Gene Lists **eTable 21.** Candidate Genes Proposed to Harbor Risk Variants Associated With Risk for Viral Respiratory Failure **eTable 22.** Capture Kits Used in Whole Exome Sequencing Generation **eFigure 1.** Geographic Ancestry and Clustering for Combined Cohort **eFigure 2.** Ultra-Rare Synonymous Model Quantile-Quantile Plot for Combined Cohort **eFigure 3.** Ultra-Rare Synonymous Model Quantile-Quantile Plot for Unresolved Cohort **eFigure 4.** Ultra-Rare Synonymous Model Quantile-Quantile Plot for Respiratory Failure Cohort **eFigure 5.** Ultra-Rare Loss-of-Function Model Quantile-Quantile Plot for Combined Cohort **eFigure 6.** Ultra-Rare Loss-of-Function Model Quantile-Quantile Plot for Unresolved Cohort **eFigure 7.** Ultra-Rare Loss-of-Function Model Quantile-Quantile Plot for Respiratory Failure **eFigure 8.** Flex Loss-of-Function Model Quantile-Quantile Plot for Combined **eFigure 9.** Flex Loss-of-Function Model Quantile-Quantile Plot for Unresolved Cohort **eFigure 10.** Flex Loss-of-Function Model Quantile-Quantile Plot for Respiratory Failure Cohort **eMethods.** Supplemental Methods
eReferences
