# Supplementary material for: Risk Variants in the Exomes of Children With Critical Illness
Source: JAMA Netw Open. 2022 Oct 28;5(10):e2239122. doi: 10.1001/jamanetworkopen.2022.39122 (PMC9617179; doi:10.1001/jamanetworkopen.2022.39122)
Supplement: Supplement. — eTable 1. Probands recruited from the Office of the Chief Medical Examiner of New York City (OCME) eTable 2. Summary of MSCH Respiratory Failure Cohort eTable 3. Collapsing Model Qualifying Variant Definition eTable 4. Genes Associated With Viral Respiratory Failure eTable 5. Summary of Diagnostic Variants eTable 6. Top Human Phenotype Ontology Terms eTable 7. Samples Removed During Data Cleaning eTable 8. Top 10 Genes in Ultra-Rare Synonymous Model in Combined Cohort eTable 9. Top 10 Genes in Ultra-Rare Synonymous Model in Unresolved Cohort eTable 10. Top 10 Genes in Ultra-Rare Synonymous Model in Respiratory Failure Cohort eTable 11. Top 10 Genes in Ultra-Rare Loss-of-Function Model in Combined Cohort eTable 12. Top 10 Genes in Ultra-Rare Loss-of-Function Model in Unresolved Cohort eTable 13. Top 10 Genes in Ultra-Rare Loss-of-Function Model in Respiratory Failure Cohort eTable 14. Top 10 Genes in Flex Loss-of-Function Model in Combined Cohort eTable 15. Top 10 Genes in Flex Loss-of-Function Model in Unresolved Cohort eTable 16. Top 10 Genes in Flex Loss-of-Function Model in Respiratory Failure Cohort eTable 17. Data for Gene-Set Enrichment Analysis for Combined Cohort eTable 18. Loss-of-Function Burden in Children With Critical Illness but Without a Causative Genetic Diagnosis eTable 19. Loss-of-Function Burden in Children With Viral Respiratory Failure in Genes With and Without a Known Disease Association eTable 20. Loss-of-Function Burden in Children With Viral Respiratory Failure in Immunodeficiency and Primary Ciliary Dyskinesia Gene Lists eTable 21. Candidate Genes Proposed to Harbor Risk Variants Associated With Risk for Viral Respiratory Failure eTable 22. Capture Kits Used in Whole Exome Sequencing Generation eFigure 1. Geographic Ancestry and Clustering for Combined Cohort eFigure 2. Ultra-Rare Synonymous Model Quantile-Quantile Plot for Combined Cohort eFigure 3. Ultra-Rare Synonymous Model Quantile-Quantile Plot for Unresolved Cohort eFigure 4. Ultra-R [file jamanetwopen-e2239122-s001.pdf]

Supplementary Online Content

Motelow JE, Lippa NC, Hostyk J, et al. Risk variants in the exomes of children with critical illness. *JAMA Netw Open*. 2022;5(10):e2239122. doi:10.1001/jamanetworkopen.2022.39122

eTable 1. Probands recruited from the Office of the Chief Medical Examiner of New York City (OCME)
eTable 2. Summary of MSCH Respiratory Failure Cohort
eTable 3. Collapsing Model Qualifying Variant Definition
eTable 4. Genes Associated With Viral Respiratory Failure
eTable 5. Summary of Diagnostic Variants
eTable 6. Top Human Phenotype Ontology Terms
eTable 7. Samples Removed During Data Cleaning
eTable 8. Top 10 Genes in Ultra-Rare Synonymous Model in Combined Cohort
eTable 9. Top 10 Genes in Ultra-Rare Synonymous Model in Unresolved Cohor
eTable 10. Top 10 Genes in Ultra-Rare Synonymous Model in Respiratory Failure Cohort
eTable 11. Top 10 Genes in Ultra-Rare Loss-of-Function Model in Combined Cohort
eTable 12. Top 10 Genes in Ultra-Rare Loss-of-Function Model in Unresolved Cohort
eTable 13. Top 10 Genes in Ultra-Rare Loss-of-Function Model in Respiratory Failure Cohort
eTable 14. Top 10 Genes in Flex Loss-of-Function Model in Combined Cohort
eTable 15. Top 10 Genes in Flex Loss-of-Function Model in Unresolved Cohort
eTable 16. Top 10 Genes in Flex Loss-of-Function Model in Respiratory Failure Cohort
eTable 17. Data for Gene-Set Enrichment Analysis for Combined Cohort
eTable 18. Loss-of-Function Burden in Children With Critical Illness but Without a Causative Genetic Diagnosis
eTable 19. Loss-of-Function Burden in Children With Viral Respiratory Failure in Genes With and Without a Known Disease Association
eTable 20. Loss-of-Function Burden in Children With Viral Respiratory Failure in Immunodeficiency and Primary Ciliary Dyskinesia Gene Lists
eTable 21. Candidate Genes Proposed to Harbor Risk Variants Associated With Risk for Viral Respiratory Failure
eTable 22. Capture Kits Used in Whole Exome Sequencing Generation
eFigure 1. Geographic Ancestry and Clustering for Combined Cohort
eFigure 2. Ultra-Rare Synonymous Model Quantile-Quantile Plot for Combined Cohort
eFigure 3. Ultra-Rare Synonymous Model Quantile-Quantile Plot for Unresolved Cohort
eFigure 4. Ultra-Rare Synonymous Model Quantile-Quantile Plot for Respiratory Failure Cohort
eFigure 5. Ultra-Rare Loss-of-Function Model Quantile-Quantile Plot for Combined Cohort
eFigure 6. Ultra-Rare Loss-of-Function Model Quantile-Quantile Plot for Unresolved Cohort
eFigure 7. Ultra-Rare Loss-of-Function Model Quantile-Quantile Plot for Respiratory Failure
eFigure 8. Flex Loss-of-Function Model Quantile-Quantile Plot for Combined
eFigure 9. Flex Loss-of-Function Model Quantile-Quantile Plot for Unresolved Cohort
eFigure 10. Flex Loss-of-Function Model Quantile-Quantile Plot for Respiratory Failure Cohort
eMethods. Supplemental Methods
eReferences

This supplementary material has been provided by the authors to give readers additional information about their work.

© 2022 Motelow JE et al. *JAMA Network Open.*

eTable 1. *Probands recruited from the Office of the Chief Medical Examiner of New York City (OCME)*

ID	***Age in Months***	***Sequence Prediction of Geographic Descent***	***Immediate Cause of Death***	***Adequate Ancestry Matched in Combined Cohort***	***Viral Respiratory Failure Cohort***	***Reason for exclusion from Respiratory Failure Cohort***	***Adequate Ancestry Match in Viral Respiratory Failure Cohort***
O1	4	Latino	Viral Bronchiolitis	X	X		X
O2	2	African	Bronchiolitis and Interstitial Pneumonia, (Probable Viral)	X	X		X
O3	3	African	Acute Bronchiolitis and Early Bronchopneumonia due to Prematurity	X		“Prematurity”	X
O4	0	African	Bronchiolitis and Interstitial Pneumonia, (Probable Viral)	X	X		X
O5	3	African	Bronchiolitis and Interstitial, Viral - Type, Pneumonia	X	X		X
O6	1	Latino	Acute bronchopneumonia and bronchiolitis complicating interstitial pneumonia, (probable viral etiology)	X	X		X
O7	3	Latino	Upper Respiratory Tract Infection, Probably Viral, Complicated By Bronchitis And Bronchiolitis	X	X		X
O8	10	African	Klebsiella Bronchopneumonia Complicating Bronchiolitis	X	X		X

© 2022 Motelow JE et al. *JAMA Network Open.*

ID	***Age in Months***	***Sequence Prediction of Geographic Descent***	***Immediate Cause of Death***	***Adequate Ancestry Matched in Combined Cohort***	***Viral Respiratory Failure Cohort***	***Reason for exclusion from Respiratory Failure Cohort***	***Adequate Ancestry Match in Viral Respiratory Failure Cohort***
O9	12	African	Chronic laryngitis, tracheitis, bronchitis and bronchiolitis due to probable viral infection. Chronic bronchial asthma and anomalous R coronary artery origin at left sinus w/ acutely angled takeoff	X		"Chronic laryngitis"	X
O10	1	African	Respiratory syncytial virus bronchiolitis	X	X		X
O11	1	South Asian	Acute viral bronchiolitis with bronchopneumonia	X	X		
O12	0	East Asian	Adenoviral bronchiolitis		X		
O13	11	African	Chronic bronchiolitis	X		"Chronic"	X
O14	11	East Asian	Complications of Parainfluenza 3 viral infection including myocarditis,			"myocarditis"	
O15	22	European	Acute Bronchopneumonia complicating Tracheitis, Bronchitis and Bronchiolitis of Probable Viral Etiology		X		
O16	14	African	Bronchiolitis of probable viral origin	X	X		X
O17	5	East Asian	Acute Bronchiolitis		X		
O18	2	African	Bronchiolitis and Pneumonitis	X	X		X

Summary of limited clinical information available from the OCME and inclusion in analysis. We generated probability estimates for each of six geographic ancestry groups (European, African, Latino, East Asian, South Asian and Middle Eastern) for each sample using a neural-network that was pre-trained on samples with known ancestry. We used a 90% probability cut-off to assign a

© 2022 Motelow JE et al. *JAMA Network Open.*

predicted geographic ancestry for each sample. This prediction is in column “Sequence Prediction of Geographic Descent”. “Adequate Ancestry Matched in Combined Cohort” indicates membership in an included cluster in the combined analysis (see eFigure 1A-C). “Viral Respiratory Failure Cohort” cohort indicates inclusion in this cohort. Probands with evidence of chronic illness prior to viral respiratory infection were excluded from viral respiratory failure cohort. Reasoning is provided in the column “Reason for exclusion from Respiratory Failure Cohort”. “Adequate Ancestry Match in Viral Respiratory Failure Cohort” indicates membership in an included cluster in the viral respiratory failure cohort (see eFigure 1G-I).

© 2022 Motelow JE et al. *JAMA Network Open.*

eTable 2. *Summary of MSCH Respiratory Failure Cohort.*

ID	Virus	Respiratory Support	Analyzed Cluster
1	respiratory syncytial virus	CMV	x
2	respiratory syncytial virus	CMV	
3	human rhinovirus/enterovirus	BPAP	x
4	influenza A H1 2009 virus	BPAP	x
5	respiratory syncytial virus	CMV	
6	human rhinovirus/enterovirus, respiratory syncytial virus	BPAP	x
7	respiratory syncytial virus	CPAP	
8	human rhinovirus/enterovirus	BPAP	x
9	human rhinovirus/enterovirus	CMV	x
10	adenovirus, human metapneumovirus	HFOV	
11	human rhinovirus/enterovirus	BPAP	x
12	human rhinovirus/enterovirus	CPAP	x
13	human rhinovirus/enterovirus	HFOV	x
14	human rhinovirus/enterovirus	CMV	
15	human rhinovirus/enterovirus	BPAP	x
16	human rhinovirus/enterovirus	CMV	
17	human rhinovirus/enterovirus, respiratory syncytial virus	BPAP	x
18	respiratory syncytial virus	CMV	
19	respiratory syncytial virus	BPAP	
20	human rhinovirus/enterovirus	CPAP	x
21	respiratory syncytial virus	BPAP	x
22	respiratory syncytial virus	CMV	x

Summary of 22 probands included in the viral respiratory failure cohort enrolled at Morgan Stanley Children's Hospital of NewYork-Presbyterian (MSCH) – Columbia University Irving Medical Center (CUIMC). Viral etiology extracted from medical record. "Respiratory support" refers to maximum respiratory support during the admission meeting study inclusion criteria. BPAP = bilevel positive airway pressure, CPAP = continuous positive airway pressure, CMV = conventional mechanical ventilation, HFOV = high frequency oscillatory ventilation.

© 2022 Motelow JE et al. *JAMA Network Open.*

eTable 3. *Collapsing Model Qualifying Variant Definition*

Model Name	***Included Effects***	***GnomAD Exome Max AF Population Specific***	***GnomAD Genome Max AF Global***	***Cohorts Analyzed Independently with Model***
Ultra-Rare Synonymous	Synonymous	0	0	Combined, Unresolved, Viral Respiratory Failure
Ultra-Rare LOF	LOF	0	0	Combined, Unresolved, Viral Respiratory Failure
Flex LOF	LOF	0.001	0.001	Combined, Unresolved, Viral Respiratory Failure

Summary of collapsing models and qualifying variant definitions. LOF = predicted loss-of-function effect. LOF effects include stop gained, frameshift, splice acceptor, and splice donor variants.

© 2022 Motelow JE et al. *JAMA Network Open.*

eTable 4. *Genes Associated with Viral Respiratory Failure*

Gene
STAT2
IRF3
TBK1
IFNAR1
TLR3
IFNAR2
IRF7
IRF9
STAT1
TICAM1
TRAF3
UNC93B1
IKBKG

Viral immunodeficiency gene list was drawn from [1].

© 2022 Motelow JE et al. *JAMA Network Open.*

eTable 5. Summary of Diagnostic Variants

ID[d]	Gene	Genomic Location in GRCh37[e,f]	Zygosity	Inherited or De Novo	Variant Effect	ACMG/AMP Clinical Classification of Variant	Source of ACMG/AMP Classification[a]	Case Status	MIM Link
1	*ACSF3*	16-89169034-G-A	Compound Heterozygous	Inherited	Stop Gained	Not Done[b]	n/a	Resolved	https://omim.org/entry/614245
1	*ACSF3*	16-89199569-AG-A	Compound Heterozygous	Inherited	Frameshift	Not Done[b]	n/a	Resolved	https://omim.org/entry/614245
2	*AKT3*	1-243716229-T-C	Mosaic Heterozygous	De Novo	Missense	Likely pathogenic	CLIA	Resolved	https://omim.org/entry/611223
3	*CHD8*	14-21871373-T-C	Heterozygous	Inherited	Splice site acceptor	Likely pathogenic	CLIA	Resolved	https://omim.org/entry/610528
4	*CHST14*	15-40764278-G-A	Homozygous	Inherited	Missense	Likely pathogenic	CLIA	Resolved	https://omim.org/entry/608429
5	*COL4A1*	13-110830200-G-C	Heterozygous	De Novo	Missense	Pathogenic	CLIA	Resolved	https://omim.org/entry/120130

© 2022 Motelow JE et al. *JAMA Network Open.*

ID[d]	Gene	Genomic Location in GRCh37[e,f]	Zygosity	Inherited or De Novo	Variant Effect	ACMG/AMP Clinical Classification of Variant	Source of ACMG/AMP Classification[a]	Case Status	MIM Link
6	*CSNK2B*	6-31634633-TG-T	Heterozygous	Unknown	Frameshift	Deleterious variant in a gene of uncertain significance	CLIA	Resolved	https://omim.org/entry/115441
7	*DEPDC5*	22-32269315-C-CCT	Heterozygous	Inherited	Frameshift	Likely pathogenic	CLIA	Resolved	https://omim.org/entry/614191
8	*DGUOK*	2-74177731-CT-A	Homozygous	Inherited	Frameshift	Pathogenic	CLIA	Resolved	https://omim.org/entry/601465
9	*DNMT3A*	2-25497921-C-T	Heterozygous	De Novo	Stop Gained	Pathogenic	CLIA	Resolved	https://omim.org/entry/602769
10	*DOCK6*	19-11338160-G-C	Homozygous	Inherited	Splice region variant	VUS	CLIA	Resolved	https://omim.org/entry/614194
11	*EBF3*	10-131639213-CTG-C	Heterozygous	Unknown	Frameshift	Likely pathogenic	CLIA	Resolved	https://omim.org/entry/607407
12	*ECEL1*	2-233348162-C-T	Compound Heterozygous	Inherited	Stop Gained	Pathogenic	CLIA	Resolved	https://omim.org/entry/605896

© 2022 Motelow JE et al. *JAMA Network Open.*

ID[d]	Gene	Genomic Location in GRCh37[e,f]	Zygosity	Inherited or De Novo	Variant Effect	ACMG/AMP Clinical Classification of Variant	Source of ACMG/AMP Classification[a]	Case Status	MIM Link
12	*ECEL1*	2-233348866-G-A	Compound Heterozygous	Inherited	Missense	Likely pathogenic	CLIA	Resolved	https://omim.org/entry/605896
13	*ELN*	7-73478001-GT-G	Heterozygous	De Novo	Frameshift	Likely pathogenic	CLIA	Resolved	https://omim.org/entry/130160
14	*EPCAM*	2-47604159-T-TC	Homozygous	Inherited	Frameshift	Not Done[b]	n/a	Partially Resolved	https://omim.org/entry/185535
15	*FBN1*	15-48780630-A-G	Heterozygous	Unknown	Missense	Pathogenic	CLIA	Resolved	https://omim.org/entry/134797
16	*FGFR1*	8-38275745-C-T	Heterozygous	De Novo	Splice site donor	Pathogenic	CLIA	Resolved	https://omim.org/entry/136350
17	*FGFR2*	10-123276865-G-C	Heterozygous	De Novo	Missense	Pathogenic	CLIA	Partially Resolved	https://omim.org/entry/176943
18	*FLG*	1-152284040-CT-C	Heterozygous	Inherited	Frameshift	Likely pathogenic	CLIA	Resolved	https://omim.org/entry/135940

© 2022 Motelow JE et al. *JAMA Network Open.*

ID[d]	Gene	Genomic Location in GRCh37[e,f]	Zygosity	Inherited or De Novo	Variant Effect	ACMG/AMP Clinical Classification of Variant	Source of ACMG/AMP Classification[a]	Case Status	MIM Link
19	*FLT4*	5-180038393-GTGTAGGGC-G	Heterozygous	Inherited	Frameshift	Likely pathogenic	CLIA	Resolved	https://omim.org/entry/136352
20	*HNF1B*	17-36099569-G-A	Heterozygous	Inherited	Stop Gained	Pathogenic	CLIA	Partially Resolved	https://omim.org/entry/189907
21	*KCNH1*	1-210977463-T-C	Heterozygous	De Novo	Missense	Pathogenic	CLIA	Resolved	https://omim.org/entry/603305
22	*KCNT1*	9-138667192-C-G	Heterozygous	De Novo	Missense	Pathogenic	CLIA	Resolved	https://omim.org/entry/608167
23	*KIF11*	10-94409740-T-TC	Heterozygous	Inherited	Frameshift	Likely pathogenic	CLIA	Resolved	https://omim.org/entry/148760
24	*KMT2A*	11-118344525-A-AT	Heterozygous	De Novo	Frameshift	Pathogenic	CLIA	Resolved	https://omim.org/entry/159555
25	*KMT2D*	12-49426187-G-A	Heterozygous	Unknown	Stop Gained	Likely pathogenic	CLIA	Resolved	https://omim.org/entry/602113

© 2022 Motelow JE et al. *JAMA Network Open.*

ID[d]	Gene	Genomic Location in GRCh37[e,f]	Zygosity	Inherited or De Novo	Variant Effect	ACMG/AMP Clinical Classification of Variant	Source of ACMG/AMP Classification[a]	Case Status	MIM Link
25	*KMT2D*	12-49444757-AG-A	Heterozygous	Unknown	Frameshift	Likely pathogenic	CLIA	Resolved	https://omim.org/entry/602113
26	*MC4R*	18-58038687-G-T	Heterozygous	Unknown	Missense	Pathogenic	CLIA	Partially Resolved	https://omim.org/entry/155541
27	*MSH6*	2-48018067-TG-T	Homozygous	Inherited	Frameshift	Likely pathogenic	CLIA	Resolved	https://omim.org/entry/600678
28	*MTOR*	1-11184573-G-T	Mosaic Heterozygous	De Novo	Missense	Pathogenic	CLIA	Resolved	https://omim.org/entry/601231
29	*NDUFS4*	5-52954385-G-C	Homozygous	Inherited	Missense	Likely pathogenic	CLIA	Resolved	https://omim.org/entry/602694
30	*NSD1*	5-176700744-C-T	Heterozygous	Unknown	Stop Gained	Pathogenic	CLIA	Resolved	https://omim.org/entry/606681
31	*PAFAH1B1*	17-2573504-T-A	Heterozygous	De Novo	Missense	Likely pathogenic	CLIA	Resolved	https://omim.org/entry/601545

© 2022 Motelow JE et al. *JAMA Network Open.*

ID[d]	Gene	Genomic Location in GRCh37[e,f]	Zygosity	Inherited or De Novo	Variant Effect	ACMG/AMP Clinical Classification of Variant	Source of ACMG/AMP Classification[a]	Case Status	MIM Link
32	*PAFAH1B1*	17-2583522-G-A	Heterozygous	De Novo	Missense	VUS	CLIA	Resolved	https://omim.org/entry/601545
33	*PCDH19*	X-99662647-G-A	Heterozygous	Unknown	Stop Gained	Likely pathogenic	CLIA	Resolved	https://omim.org/entry/300460
34	*PDHA1*	x-19377756-T-TAAGTCA	Hemizygous	Inherited	In Frame Insertion	VUS	CLIA	Resolved	https://omim.org/entry/300502
35	*PIGW*	17-34893056-A-G	Compound Heterozygous	Inherited	Missense	VUS	CLIA	Resolved	https://omim.org/entry/610275
35	*PIGW*	17-34893720-C-T	Compound Heterozygous	Inherited	Missense	VUS	CLIA	Resolved	https://omim.org/entry/610275
36	*PPT1*	1-40562790-TCCCATG-T	Homozygous	Inherited	In Frame Deletion	Likely pathogenic	CLIA	Resolved	https://omim.org/entry/600722

© 2022 Motelow JE et al. *JAMA Network Open.*

ID[d]	Gene	Genomic Location in GRCh37[e,f]	Zygosity	Inherited or De Novo	Variant Effect	ACMG/AMP Clinical Classification of Variant	Source of ACMG/AMP Classification[a]	Case Status	MIM Link
37	*PROS1*	3-93624643-T-C	Heterozygous	Unknown	Missense	Risk Allele	CLIA	Partially Resolved	https://omim.org/entry/176880
38	*SCN1A*	2-166848542-GGGT-G	Heterozygous	De Novo	In Frame Deletion	VUS	CLIA	Resolved	https://omim.org/entry/182389
39	*SCN1A*	2-166892692-C-A	Mosaic Heterozygous	Unknown	Stop Gained	Likely pathogenic	CLIA	Resolved	https://omim.org/entry/182389
40	*SCN1A*	2-166895942-TGAAC-T	Heterozygous	Evidence of parental mosaicism	Frameshift	Pathogenic	CLIA	Resolved	https://omim.org/entry/182389
18	*SCN2A*	2-166165703-A-G	Heterozygous	De Novo	Missense	Pathogenic	CLIA	Resolved	https://omim.org/entry/182390
41	*SCN2A*	2-166198975-G-A	Heterozygous	De Novo	Missense	Likely pathogenic	CLIA	Resolved	https://omim.org/entry/182390
34	*SCN2A*	2-166243470-A-G	Heterozygous	Inherited	Missense	VUS	CLIA	Resolved	https://omim.org/entry/182390

© 2022 Motelow JE et al. *JAMA Network Open.*

ID[d]	Gene	Genomic Location in GRCh37[e,f]	Zygosity	Inherited or De Novo	Variant Effect	ACMG/AMP Clinical Classification of Variant	Source of ACMG/AMP Classification[a]	Case Status	MIM Link
42	*SCN2A*	2-166245961-G-T	Heterozygous	De Novo	Missense	Pathogenic	CLIA	Resolved	https://omim.org/entry/182390
43	*SMARCB1*	22-24176330-G-A	Heterozygous	De Novo	Missense	Likely pathogenic	CLIA	Resolved	https://omim.org/entry/601607
44	*SPAST*	2-32361636-GAGA-G	Heterozygous	De Novo	Frameshift	Likely pathogenic	CLIA	Resolved	https://omim.org/entry/604277
17	*TGFBR2*	3-30729962-C-T	Heterozygous	De Novo	Missense	Pathogenic	CLIA	Partially Resolved	https://omim.org/entry/190182
45	*TNNT2*	1-201331098-ATCT-A	Heterozygous	Inherited	In Frame Deletion	Pathogenic	CLIA	Partially Resolved	https://omim.org/entry/191045
1	*TOP3A*	17-18198015-A-ACC	Compound Heterozygous	Inherited	Splice site donor	Likely pathogenic	CLIA	Resolved	https://omim.org/entry/601243
1	*TOP3A*	17-18206010-G-A	Compound Heterozygous	Inherited	Missense	VUS	CLIA	Resolved	https://omim.org/entry/601243

© 2022 Motelow JE et al. *JAMA Network Open.*

ID[d]	Gene	Genomic Location in GRCh37[e,f]	Zygosity	Inherited or De Novo	Variant Effect	ACMG/AMP Clinical Classification of Variant	Source of ACMG/AMP Classification[a]	Case Status	MIM Link
46	*TP53*	17-7578404-A-G	Mosaic Heterozygous	De Novo	Missense	Pathogenic	CLIA	Resolved	https://omim.org/entry/191170
47	*TP63*	3-189586404-G-A	Heterozygous	De Novo	Missense	Not Done[b]	n/a	Resolved	https://omim.org/entry/603273
48	*COL4A4*	2-227966616-C-A	Compound Heterozygous	Inherited	Missense	Likely pathogenic	CLIA	Partially Resolved	https://omim.org/entry/120131
48	*COL4A4*	17-29556167-G-A	Compound Heterozygous	Unknown	Missense	Pathogenic	CLIA	Partially Resolved	https://omim.org/entry/120131
49	*PIGW*	17-34893056-A-G	Compound Heterozygous	Inherited	Missense	VUS	CLIA	Resolved	https://omim.org/entry/610275
49	*PIGW*	17-34893720-C-T	Compound Heterozygous	Inherited	Missense	VUS	CLIA	Resolved	https://omim.org/entry/610275
50	*OTX2*	14-57268813-G-T	Heterozygous	De Novo	Stop Gained	Likely pathogenic	CLIA	Resolved	https://omim.org/entry/600037

© 2022 Motelow JE et al. *JAMA Network Open.*

ID[d]	Gene	Genomic Location in GRCh37[e,f]	Zygosity	Inherited or De Novo	Variant Effect	ACMG/AMP Clinical Classification of Variant	Source of ACMG/AMP Classification[a]	Case Status	MIM Link
51	*MCM3AP*	21-47692533-C-T	Heterozygous	Inherited	Missense	VUS	CLIA	Resolved	https://omim.org/entry/603294
51	*MCM3AP*	21-47705041-G-A	Heterozygous	Inherited	Stop Gained	Likely pathogenic	CLIA	Resolved	https://omim.org/entry/603294

Table summarizing the diagnostic variants of the children whose phenotypes are fully (n = 46) or partially (n = 11) by the genetic finding.

[a] ACMG/AMP classification of variants was either performed in a CLIA approved laboratory when the sanger confirmation was ordered or in the research laboratory. ACMG/AMP classifications were not routinely updated overtime using updated guidelines. Rather at the time of analysis, the most recent classification guidelines were used.

[b] CLIA Sanger confirmed but classification not done by CLIA lab

[c] CLIA Sanger confirmed but classification not done by CLIA lab

[d] Study ID bears no relationship to any coded or uncoded data. Subject ID is assigned based on the alphabetical order of the genes and is used to show if multiple variants are from the same proband.

[e] Some data presented in this table is also published in Lippa et. al. Genet Med. 2022 Apr;24(4):862-869. doi: 10.1016/j.gim.2021.12.010. PMID: 35078725.

[f] Six subjects had a diagnosis from non-ES genetic testing that either partially (n = 3) or fully (n = 3) explained the subjects phenotype.

© 2022 Motelow JE et al. *JAMA Network Open.*

eTable 6. *Top Human Phenotype Ontology Terms*

HPO Label	*HPO ID*	*Count*
Respiratory failure	HP:0002878	110
Seizure	HP:0001250	108
Severe viral infection	HP:0031691	76
Fever	HP:0001945	69
Respiratory distress	HP:0002098	69
Hypoxemia	HP:0012418	50
Tube feeding	HP:0033454	49
Respiratory failure requiring assisted ventilation	HP:0004887	47
Feeding difficulties	HP:0011968	47
Cough	HP:0012735	45

Top ten human phenotype ontology (HPO) terms describing the combined MSCH and OCME cohorts. Data drawn from admission notes.

© 2022 Motelow JE et al. *JAMA Network Open.*

eTable 7. *Samples Removed During Data Cleaning*

**Cohort**	**Initial Cohort**	**After QC**	**After Relatedness Check**	**Cases Included in Analyzed Clusters**	**Controls Included in Analyzed Clusters**
All	285	282	280	231	5322
Unresolved	229	226	225	176	5180
Respiratory Failure	36	36	36	25	2973

Table summarizing samples removed at each quality control (QC) step. Final QC step is to include only those samples in ancestry-matched clusters with adequate sample size.

© 2022 Motelow JE et al. *JAMA Network Open.*

eTable 8. *Top 10 Genes in Ultra-Rare Synonymous Model in Combined Cohort*

Gene	P-Value[a]	Estimate[a]	Conf Low[a]	Conf High[a]	Case w/ QV[b]	Case w/o QV[c]	Control w/ QV[b]	Control w/o QV[c]	O[d]
TAF6	1.9E-05	Inf	15.78751	Inf	3	228	0	5322	X
NUDT12	0.000728	35.76801	3.885492	448.9256	3	228	2	5320	
BBS10	0.000766	38.3935	3.869727	511.0471	3	228	2	5320	X
SNRNP200	0.001003	Inf	5.859329	Inf	2	229	0	5322	X
MUL1	0.001697	Inf	4.564471	Inf	2	229	0	5322	
CPT1A	0.001938	Inf	4.089683	Inf	2	229	0	5322	X
FUT3	0.001938	Inf	4.089683	Inf	2	229	0	5322	
TUSC1	0.002058	Inf	3.965641	Inf	2	229	0	5322	
PIKFYVE	0.002848	8.708613	1.950436	31.10472	4	227	10	5312	X
GPR150	0.00328	Inf	3.131536	Inf	2	229	0	5322	

Summary of top 10 genes in ultra-rare synonymous model in combined cohort. None achieved study wide significance.
[a] Pooled odds ratio, 95% confidence intervals and false discovery rate corrected *P*-values were generated from the exact two-sided Cochran-Mantel-Haenszel (CMH) test.
[b] "w/ QV" refers to the number of cases or controls with a qualifying variant. See eTable 3 for model parameters.
[c] "w/o QV" refers to the number of cases or controls without a qualifying variant. See eTable 3 for model parameters.
[d] "O" refers to genes with a disease association. See eMethods.

© 2022 Motelow JE et al. *JAMA Network Open.*

eTable 9. *Top 10 Genes in Ultra-Rare Synonymous Model in Unresolved Cohort*

Gene	P-Value[a]	Estimate[a]	Conf Low[a]	Conf High[a]	Case w/ QV[b]	Case w/o QV[c]	Control w/ QV[b]	Control w/o QV[c]	O[d]
'TAF6'	8.13E-06	Inf	21.77756	Inf	3	173	0	5180	X
'PIKFYVE'	0.001422	10.65313	2.378833	38.21467	4	172	10	5170	X
'TUSC1'	0.001826	Inf	4.22085	Inf	2	174	0	5180	
'DNMBP'	0.002369	76.82831	3.59182	4836.768	2	174	1	5179	X
'PKP1'	0.002923	14.64621	2.280798	72.44395	3	173	6	5174	X
'STRADA'	0.003428	44.91609	2.930886	695.0042	2	174	2	5178	X
'TYW5'	0.005263	27.60767	2.317788	211.4078	2	174	4	5176	
'LZTS1'	0.005263	27.60767	2.317788	211.4078	2	174	4	5176	X
'EGR4'	0.005388	31.11325	2.302868	309.4922	2	174	3	5177	
'SLCO4C1'	0.005899	32.41464	2.196775	478.9045	2	174	2	5178	

Summary of top 10 genes in ultra-rare synonymous model in unresolved cohort. None achieved study wide significance.
[a] Pooled odds ratio, 95% confidence intervals and false discovery rate corrected *P*-values were generated from the exact two-sided Cochran-Mantel-Haenszel (CMH) test.
[b] “w/ QV” refers to the number of cases or controls with a qualifying variant. See eTable 3 for model parameters.
[c] “w/o QV” refers to the number of cases or controls without a qualifying variant. See eTable 3 for model parameters.
[d] “O” refers to genes with a disease association. See eMethods.

© 2022 Motelow JE et al. *JAMA Network Open.*

eTable 10. *Top 10 Genes in Ultra-Rare Synonymous Model in Respiratory Failure Cohort*

Gene	P-Value[a]	Estimate[a]	Conf Low[a]	Conf High[a]	Case w/ QV[b]	Case w/o QV[c]	Control w/ QV[b]	Control w/o QV[c]	O[d]
'MAN1C1'	0.000199	251.44	12.67	13242.04	2	23	1	2972	
'WDR7'	0.000658	84.69	6.78	769.62	2	23	3	2970	
'PLXNB1'	0.001367	51.00	4.64	330.81	2	23	5	2968	X
'SLIT3'	0.002319	36.52	3.52	207.25	2	23	7	2966	
'ADAMTS15'	0.008339	Inf	3.05	Inf	1	24	0	2973	
'CA1'	0.008339	Inf	3.05	Inf	1	24	0	2973	
'TBX21'	0.008339	Inf	3.05	Inf	1	24	0	2973	X
'MBNL1'	0.008339	Inf	3.05	Inf	1	24	0	2973	
'SLC22A6'	0.008339	Inf	3.05	Inf	1	24	0	2973	
'PMM2'	0.008339	Inf	3.05	Inf	1	24	0	2973	X

Summary of top 10 genes in ultra-rare synonymous model in respiratory failure cohort. None achieved study wide significance.
[a] Odds ratio, 95% confidence intervals and false discovery rate corrected *P*-values were generated from the Fisher's exact test.
[b] “w/ QV” refers to the number of cases or controls with a qualifying variant. See eTable 3 for model parameters.
[c] “w/o QV” refers to the number of cases or controls without a qualifying variant. See eTable 3 for model parameters.
[d] “O” refers to genes with a disease association. See eMethods.

© 2022 Motelow JE et al. *JAMA Network Open.*

eTable 11. *Top 10 Genes in Ultra-Rare Loss-of-Function Model in Combined Cohort*

Gene	P-Value[a]	Estimate[a]	Conf Low[a]	Conf High[a]	Case w/ QV[b]	Case w/o QV[c]	Control w/ QV[b]	Control w/o QV[c]	O[d]
'ULK2'	0.000662	Inf	7.166405	Inf	2	229	0	5322	
'SCN1A'	0.001065	Inf	5.692446	Inf	2	229	0	5322	X
'PP2D1'	0.002906	Inf	3.297654	Inf	2	229	0	5322	
'TANC1'	0.00689	39.81628	2.034892	2345.863	2	229	1	5321	
'SCD5'	0.006964	30.71519	2.015724	467.3302	2	229	2	5320	
'ALOX15B'	0.008005	30.14959	1.879416	471.3741	2	229	2	5320	
'PLEKHH2'	0.011975	21.26075	1.501693	299.7895	2	229	2	5320	
'AMPD1'	0.013279	21.87164	1.423043	336.6552	2	229	2	5320	X
'NDST1'	0.018507	Inf	1.359857	Inf	1	230	0	5322	X
'UQCRB'	0.018507	Inf	1.359857	Inf	1	230	0	5322	X

Summary of top 10 genes in ultra-rare loss-of-function model in respiratory failure cohort. None achieved study wide significance.
[a] Pooled odds ratio, 95% confidence intervals and false discovery rate corrected *P*-values were generated from the exact two-sided Cochran-Mantel-Haenszel (CMH) test.
[b] "w/ QV" refers to the number of cases or controls with a qualifying variant. See eTable 3 for model parameters.
[c] "w/o QV" refers to the number of cases or controls without a qualifying variant. See eTable 3 for model parameters.
[d] "O" refers to genes with a disease association. See eMethods.

© 2022 Motelow JE et al. *JAMA Network Open.*

eTable 12. *Top 10 Genes in Ultra-Rare Loss-of-Function Model in Unresolved Cohort*

Gene	P-Value[a]	Estimate[a]	Conf Low[a]	Conf High[a]	Case w/ QV[b]	Case w/o QV[c]	Control w/ QV[b]	Control w/o QV[c]	O[d]
'ULK2'	0.000432	Inf	9.006524	Inf	2	174	0	5180	
'ALOX15B'	0.001006	118.366	5.608531	7058.661	2	174	1	5179	
'SCD5'	0.003926	42.68252	2.732851	673.8784	2	174	2	5178	
'TANC1'	0.005365	45.25925	2.324912	2644.481	2	174	1	5179	
'PLEKHH2'	0.010609	22.65831	1.603224	318.8036	2	174	2	5178	
'UQCRB'	0.01265	Inf	2.001282	Inf	1	175	0	5180	X
'SP4'	0.01265	Inf	2.001282	Inf	1	175	0	5180	
'PTEN'	0.01265	Inf	2.001282	Inf	1	175	0	5180	X
'ARL5A'	0.01265	Inf	2.001282	Inf	1	175	0	5180	
'FETUB'	0.01265	Inf	2.001282	Inf	1	175	0	5180	

Summary of top 10 genes in ultra-rare loss-of-function model in unresolved cohort. None achieved study wide significance.
[a] Pooled odds ratio, 95% confidence intervals and false discovery rate corrected *P*-values were generated from the exact two-sided Cochran-Mantel-Haenszel (CMH) test.
[b] “w/ QV” refers to the number of cases or controls with a qualifying variant. See eTable 3 for model parameters.
[c] “w/o QV” refers to the number of cases or controls without a qualifying variant. See eTable 3 for model parameters.
[d] “O” refers to genes with a disease association. See eMethods.

© 2022 Motelow JE et al. *JAMA Network Open.*

eTable 13. *Top 10 Genes in Ultra-Rare Loss-of-Function Model in Respiratory Failure Cohort*

Gene	P-Value[a]	Estimate[a]	Conf Low[a]	Conf High[a]	Case w/ QV[b]	Case w/o QV[c]	Control w/ QV[b]	Control w/o QV[c]	O[d]
'AASDH'	0.008339	Inf	3.05	Inf	1	24	0	2973	
'FAM118B'	0.008339	Inf	3.05	Inf	1	24	0	2973	
'FBXO44'	0.008339	Inf	3.05	Inf	1	24	0	2973	
'GRM5'	0.008339	Inf	3.05	Inf	1	24	0	2973	X
'MKL2'	0.008339	Inf	3.05	Inf	1	24	0	2973	X
'ZNF746'	0.008339	Inf	3.05	Inf	1	24	0	2973	
'ARRDC5'	0.008339	Inf	3.05	Inf	1	24	0	2973	
'MTTP'	0.008339	Inf	3.05	Inf	1	24	0	2973	X
'LAMC3'	0.008339	Inf	3.05	Inf	1	24	0	2973	X
'RECQL'	0.008339	Inf	3.05	Inf	1	24	0	2973	

Summary of top 10 genes in ultra-rare loss-of-function model in respiratory failure cohort. None achieved study wide significance.
[a] Odds ratio, 95% confidence intervals and false discovery rate corrected *P*-value were generated from the Fisher's exact test.
[b] "w/ QV" refers to the number of cases or controls with a qualifying variant. See eTable 3 for model parameters.
[c] "w/o QV" refers to the number of cases or controls without a qualifying variant. See eTable 3 for model parameters.
[d] "O" refers to genes with a disease association. See eMethods.

© 2022 Motelow JE et al. *JAMA Network Open.*

eTable 14. *Top 10 Genes in Flex Loss-of-Function Model in Combined Cohort*

Gene	P-Value[a]	Estimate[a]	Conf Low[a]	Conf High[a]	Case w/ QV[b]	Case w/o QV[c]	Control w/ QV[b]	Control w/o QV[c]	O[d]
'ULK2'	0.000662	Inf	7.166405	Inf	2	229	0	5322	
'TANC1'	0.000733	51.42009	3.971684	2726.361	3	228	1	5321	
'SCN1A'	0.001065	Inf	5.692446	Inf	2	229	0	5322	X
'C8G'	0.001386	23.84352	3.045341	187.466	3	228	3	5319	
'KTN1'	0.002058	Inf	3.965641	Inf	2	229	0	5322	
'PIF1'	0.003068	16.07358	2.26462	92.64572	3	228	5	5317	
'B3GNT5'	0.003117	Inf	3.176194	Inf	2	229	0	5322	
'PGAM5'	0.003227	Inf	3.12308	Inf	2	229	0	5322	
'FCRLA'	0.003531	60.38019	2.911982	3703.847	2	229	1	5321	
'AP4E1'	0.003675	55.51836	2.839121	3230.256	2	229	1	5321	X

Summary of top 10 genes in ultra-rare loss-of-function model in combined cohort. None achieved study wide significance.
[a] Pooled odds ratio, 95% confidence intervals and false discovery rate corrected *P*-value were generated from the exact two-sided Cochran-Mantel-Haenszel (CMH) test.
[b] "w/ QV" refers to the number of cases or controls with a qualifying variant. See eTable 3 for model parameters.
[c] "w/o QV" refers to the number of cases or controls without a qualifying variant. See eTable 3 for model parameters.
[d] "O" refers to genes with a disease association. See eMethods.

© 2022 Motelow JE et al. *JAMA Network Open.*

eTable 15. *Top 10 Genes in Flex Loss-of-Function Model in Unresolved Cohort*

Gene	P-Value[a]	Estimate[a]	Conf Low[a]	Conf High[a]	Case w/ QV[b]	Case w/o QV[c]	Control w/ QV[b]	Control w/o QV[c]	O[d]
'ULK2'	0.000432	Inf	9.006524	Inf	2	174	0	5180	
'PIF1'	0.000669	31.24942	3.976622	215.1017	3	173	4	5176	
'C8G'	0.001063	26.69005	3.355902	214.5365	3	173	3	5177	
'KTN1'	0.001826	Inf	4.22085	Inf	2	174	0	5180	
'B3GNT5'	0.002252	Inf	3.773099	Inf	2	174	0	5180	
'PGAM5'	0.002778	Inf	3.380785	Inf	2	174	0	5180	
'CNBD2'	0.003211	14.43833	2.206273	75.5662	3	173	5	5175	
'ZFP41'	0.003926	42.68252	2.732851	673.8784	2	174	2	5178	
'SCD5'	0.003926	42.68252	2.732851	673.8784	2	174	2	5178	
'OR5K4'	0.003927	53.23573	2.740452	3087.658	2	174	1	5179	

Summary of top 10 genes in ultra-rare loss-of-function model in unresolved cohort. None achieved study wide significance.
[a] Pooled odds ratio, 95% confidence intervals and false discovery rate corrected *P*-values were generated from the exact two-sided Cochran-Mantel-Haenszel (CMH) test.
[b] "w/ QV" refers to the number of cases or controls with a qualifying variant. See eTable 3 for model parameters.
[c] "w/o QV" refers to the number of cases or controls without a qualifying variant. See eTable 3 for model parameters.
[d] "O" refers to genes with a disease association. See eMethods.

© 2022 Motelow JE et al. *JAMA Network Open.*

eTable 16. *Top 10 Genes in Flex Loss-of-Function Model in Respiratory Failure Cohort*

Gene	P-Value[a]	Estimate[a]	Conf Low[a]	Conf High[a]	Case w/ QV[b]	Case w/o QV[c]	Control w/ QV[b]	Control w/o QV[c]	O[d]
'BBX'	0.008339	Inf	3.05	Inf	1	24	0	2973	
'FAM118B'	0.008339	Inf	3.05	Inf	1	24	0	2973	
'FBXO44'	0.008339	Inf	3.05	Inf	1	24	0	2973	
'TRIM40'	0.008339	Inf	3.05	Inf	1	24	0	2973	
'GRM5'	0.008339	Inf	3.05	Inf	1	24	0	2973	X
'MKL2'	0.008339	Inf	3.05	Inf	1	24	0	2973	X
'SV2B'	0.008339	Inf	3.05	Inf	1	24	0	2973	
'ZNF768'	0.008339	Inf	3.05	Inf	1	24	0	2973	
'COCH'	0.008339	Inf	3.05	Inf	1	24	0	2973	X
'ZNF746'	0.008339	Inf	3.05	Inf	1	24	0	2973	

Summary of top 10 genes in ultra-rare loss-of-function model in unresolved cohort. None achieved study wide significance.
[a] Odds ratio, 95% confidence intervals and false discovery rate corrected *P*-values were generated from the Fisher's exact test.
[b] “w/ QV” refers to the number of cases or controls with a qualifying variant. See eTable 3 for model parameters.
[c] “w/o QV” refers to the number of cases or controls without a qualifying variant. See eTable 3 for model parameters.
[d] “O” refers to genes with a disease association. See eMethods.

© 2022 Motelow JE et al. *JAMA Network Open.*

eTable 17. *Data for Gene-Set Enrichment Analysis for Combined Cohort*

**Gene List**[a]	**Model**[b]	**Case w/ QV**[c]	**Case w/o QV**[d]	**% Case w/ QV**[c]	**Control w/ QV**[c]	**Control w/o QV**[d]	**% Control w/ QV**[c]	**Odds Ratio**[e]	**CI**[e]	***P*-Value**[e]	**FDR-Corrected *P*-Value**[e]
Intolerant Genes	Flex	152	79	65.8%	2803	2519	52.7%	1.798	[1.4 2.4]	3.3e-05	7.3e-04
Disease Association	Flex	74	157	32.0%	1314	4008	24.7%	1.483	[1.1 2.0]	7.6e-03	2.1e-02
Disease Association	Ultra-Rare	55	176	23.8%	846	4476	15.9%	1.718	[1.2 2.4]	1.1e-03	3.8e-03
Disease Association	Rare	23	208	10.0%	564	4758	10.6%	0.942	[0.6 1.5]	9.1e-01	1.0e+00
No Disease Association	Flex	111	120	48.1%	1990	3332	37.4%	1.598	[1.2 2.1]	6.0e-04	2.9e-03
No Disease Association	Ultra-Rare	77	154	33.3%	1303	4019	24.5%	1.615	[1.2 2.2]	1.2e-03	3.8e-03
No Disease Association	Rare	47	184	20.3%	908	4414	17.1%	1.243	[0.9 1.7]	2.1e-01	3.3e-01

Gene-set burden analysis to understand the source of loss-of-function association with children with critical illness. Genes with a LOEUF score ≤ 0.680. Table shows data for Figure 2A (see figure legend for details).
Abbreviations: QV = qualifying variant, % = percentage, w/ = with, w/o = without, CI = 95% confidence interval, FDR = false discovery rate
[a] Only genes with a LOEUF score ≤ 0.680 which was identified as the threshold of peak burden (see Figure 1) were considered. “Intolerant Genes” refers to all genes with a LOEUF score ≤ 0.680. “Disease Association” refers to genes with a LOEUF score ≤ 0.680 with a known disease association. “No Disease Association” refers to genes with a LOEUF score ≤ 0.680 without a known disease association.
[b] Indicates definition of qualifying variants. See eTable 3 for model parameters
[c] “w/ QV” refers to cases or controls with a qualifying variant. See eTable 3 for model parameters.
[d] “w/o QV” refers to cases or controls without a qualifying variant. See eTable 3 for model parameters.
[e] Pooled odds ratio, 95% confidence intervals, uncorrected *p*-value and FDR corrected *p*-value were generated from the exact two-sided Cochran-Mantel-Haenszel (CMH) test.

© 2022 Motelow JE et al. *JAMA Network Open.*

eTable 18. *Loss-of-Function Burden in Children with Critical Illness but without a Causative Genetic Diagnosis*

Gene List[a]	Model[b]	Case w/ QV[c]	Case w/o QV[d]	% Case w/ QV[c]	Control w/ QV[c]	Control w/o QV[d]	% Control w/ QV[c]	Odds Ratio[e]	CI[e]	*P*-Value[e]	FDR-Corrected *P*-Value[e]
Intolerant Genes	Flex	113	63	64.2%	2711	2469	52.3%	1.728	[1.3 2.4]	6.6e-04	2.9e-03
Disease Association	Flex	47	129	26.7%	1272	3908	24.6%	1.162	[0.8 1.6]	4.2e-01	5.8e-01
Disease Association	Ultra-Rare	31	145	17.6%	822	4358	15.9%	1.199	[0.8 1.8]	3.9e-01	5.7e-01
Disease Association	Rare	18	158	10.2%	542	4638	10.5%	0.978	[0.6 1.6]	1.0e+00	1.0e+00
No Disease Association	Flex	88	88	50.0%	1919	3261	37.0%	1.789	[1.3 2.5]	2.1e-04	2.3e-03
No Disease Association	Ultra-Rare	61	115	34.7%	1249	3931	24.1%	1.798	[1.3 2.5]	4.9e-04	2.9e-03
No Disease Association	Rare	38	138	21.6%	883	4297	17.0%	1.348	[0.9 2.0]	1.3e-01	2.6e-01

Gene-set burden analysis to understand the source of loss-of-function association with children with critical illness but without a causative genetic diagnosis. Genes with a LOEUF score ≤ 0.680. Table shows data for Figure 2B (see figure legend for details). Abbreviations: QV = qualifying variant, % = percentage, w/ = with, w/o = without, CI = 95% confidence interval, FDR = false discovery rate

[a] Only genes with a LOEUF score ≤ 0.680 which was identified as the threshold of peak burden (see Figure 1) were considered. "Intolerant Genes" refers to all genes with a LOEUF score ≤ 0.680. "Disease Association" refers to genes with a LOEUF score ≤ 0.680 with a known disease association. "No Disease Association" refers to genes with a LOEUF score ≤ 0.680 without a known disease association.

[b] Indicates definition of qualifying variants. See eTable 3 for model parameters

[c] "w/ QV" refers to cases or controls with a qualifying variant. See eTable 3 for model parameters.

[d] "w/o QV" refers to cases or controls without a qualifying variant. See eTable 3 for model parameters.

[e] Pooled odds ratio, 95% confidence intervals, uncorrected *p*-value and FDR corrected *p*-value were generated from the exact two-sided Cochran-Mantel-Haenszel (CMH) test.

© 2022 Motelow JE et al. *JAMA Network Open.*

eTable 19. *Loss-of-Function Burden in Children with Viral Respiratory Failure in Genes with and without a Known Disease Association*

**Gene List**[a]	**Model**[b]	**Case w/ QV**[c]	**Case w/o QV**[d]	**% Case w/ QV**[c]	**Control w/ QV**[c]	**Control w/o QV**[d]	**% Control w/ QV**[c]	**Odds Ratio**[e]	**CI**[e]	***P*-Value**[e]	**FDR-Corrected *P*-Value**[e]
Disease Association	Flex	6	19	24.0%	720	2253	24.2%	0.988	[0.3 2.6]	1.0	1.0e+00
No Disease Association	Flex	14	11	56.0%	1057	1916	35.6%	2.306	[1.0 5.6]	3.8e-02	8.4e-02
No Disease Association	Ultra-Rare	11	14	44.0%	659	2314	22.2%	2.758	[1.1 6.6]	1.5e-02	3.7e-02
No Disease Association	Rare	7	18	28.0%	500	2473	16.8%	1.923	[0.7 4.9]	1.7e-01	2.9e-01

Gene-set burden analysis to understand the source of predicted loss-of-function association with children with viral respiratory failure. Genes with a LOEUF score ≤ 0.680. Table shows data for Figure 3 (see figure legend for details). Abbreviations: QV = qualifying variant, % = percentage, w/ = with, w/o = without, CI = 95% confidence interval, FDR = false discovery rate

[a] Only genes with a LOEUF score ≤ 0.680 which was identified as the threshold of peak burden (see Figure 1) were considered. "Intolerant Genes" refers to all genes with a LOEUF score ≤ 0.680. "Disease Association" refers to genes with a LOEUF score ≤ 0.680 with a known disease association. "No Disease Association" refers to genes with a LOEUF score ≤ 0.680 without a known disease association.

[b] Indicates definition of qualifying variants. See eTable 3 for model parameters

[c] "w/ QV" refers to cases or controls with a qualifying variant. See eTable 3 for model parameters.

[d] "w/o QV" refers to cases or controls without a qualifying variant. See eTable 3 for model parameters.

[e] Odds ratio, 95% confidence intervals, uncorrected *p*-value and FDR corrected *p*-value were generated from the Fisher's exact test.

© 2022 Motelow JE et al. *JAMA Network Open.*

eTable 20. *Loss-of-Function Burden in Children with Viral Respiratory Failure in Immunodeficiency and Primary Ciliary Dyskinesia Gene Lists*

Gene List[a]	Model[b]	Case w/ QV[c]	Case w/o QV[d]	% Case w/ QV[c]	Control w/ QV[c]	Control w/o QV[d]	% Control w/ QV[c]	Odds Ratio[e]	CI[e]	*P*-Value[e]	FDR-Corrected *P*-Value[e]
Viral Pathway	Ultra-Rare	0	25	0.0%	1	2972	0.0%	0.000	[0.0 4324.6]	1.0e+00	1.0e+00
Primary Immunodeficiency	Ultra-Rare	0	25	0.0%	147	2826	4.9%	0.000	[0.0 3.1]	6.3e-01	8.2e-01
Primary Ciliary Dyskinesia	Ultra-Rare	1	24	4.0%	20	2953	0.7%	6.142	[0.1 41.7]	1.6e-01	2.9e-01
Asthma	Ultra-Rare	0	25	0.0%	25	2948	0.8%	0.000	[0.0 20.1]	1.0e+00	1.0e+00

Gene-set burden analysis of predicted loss-of-function association for children with viral respiratory failure. Gene-sets tested are associated with primary ciliary dyskinesia, immunodeficiency, asthma, and viral respiratory failure. Table shows data for Figure 3 (see figure legend for details). Abbreviations: QV = qualifying variant, % = percentage, w/ = with, w/o = without, CI = 95% confidence interval, FDR = false discovery rate

[a] "Primary Immunodeficiency" includes 412 genes drawn from the Invitae Primary Immunodeficiency Panel. "Primary Ciliary Dyskinesia" includes 31 genes drawn from the GeneDx Primary Ciliary Dyskinesia Panel. "Viral Pathway" includes 13 genes drawn from genes previously implicated in viral respiratory failure. "Asthma" includes 101 genes implicated in susceptibility to asthma. See eMethods.

[b] Indicates definition of qualifying variants. See eTable 3 for model parameters

[c] "w/ QV" refers to cases or controls with a qualifying variant. See eTable 3 for model parameters.

[d] "w/o QV" refers to cases or controls without a qualifying variant. See eTable 3 for model parameters.

[e] Odds ratio, 95% confidence intervals, uncorrected *P*-values and FDR corrected *P*-values were generated from the Fisher's exact test.

© 2022 Motelow JE et al. *JAMA Network Open.*

eTable 21. *Candidate Genes Proposed to Harbor Risk Variants Associated with Risk for Viral Respiratory Failure*

ADAMTS7
B3GNT5
CTTN
FAM118B
FEM1B
KIAA1211
RASGRP4
RSBN1L
ZNF746

Genes with LOEUF score ≤ 0.680 harboring ultra-rare loss-of-function variants in cases but not controls in the viral respiratory failure cohort.

© 2022 Motelow JE et al. *JAMA Network Open.*

eTable 22. *Capture Kits Used in Whole Exome Sequencing Generation*

**Capture Kit**	**Case**	**Ctrl**
65MB	0	542
AgilentV4	0	109
Agilentv5	0	4
AgilentV5	0	8
AgilentV5UTR	9	51
AgilentV6	0	2
IDTERPv1	198	2285
MedExome	0	5
Roche	78	6923
RocheV2	0	32

Capture kits used for exome samples.

© 2022 Motelow JE et al. *JAMA Network Open.*

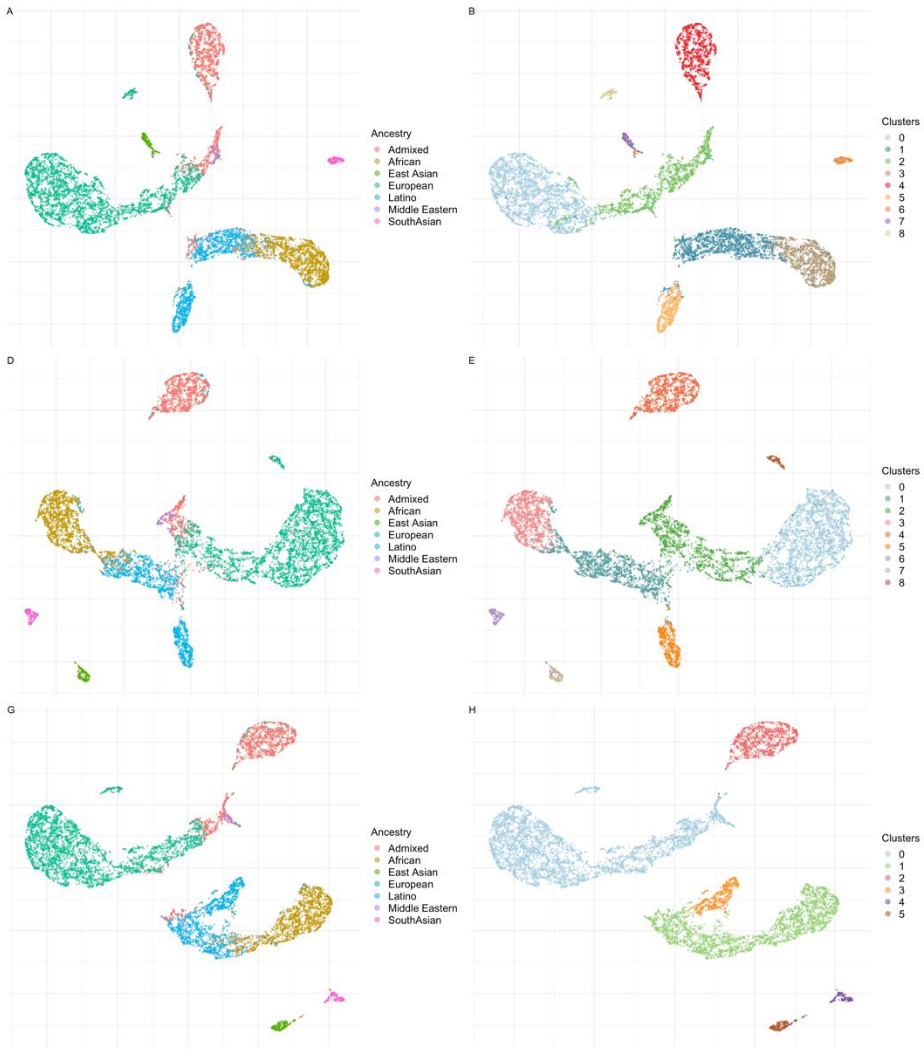

C

Cluster	Cases	Controls
0	19	2657
1	96	1676
2	29	1538
3	48	1294
4	17	1132
5	37	606
6	21	208
7	10	188
8	4	129

F

Cluster	Cases	Controls
0	15	2657
1	76	1727
2	20	1561
3	45	1272
4	13	1137
5	35	620
6	11	220
7	7	195
8	3	130

I

Cluster	Cases	Controls
0	3	4321
1	25	2973
2	2	1133
3	3	609
4	1	204
5	2	188

eFigure 1. *Geographic ancestry and clustering for combined cohort*
UMAP and cluster assignments showing geographic ancestry of case-control cohorts for the combined cohort (A-C), unresolved cohort (D-F), and viral respiratory failure cohort (G-I). Clusters shaded in red were excluded from the analysis.

© 2022 Motelow JE et al. *JAMA Network Open.*

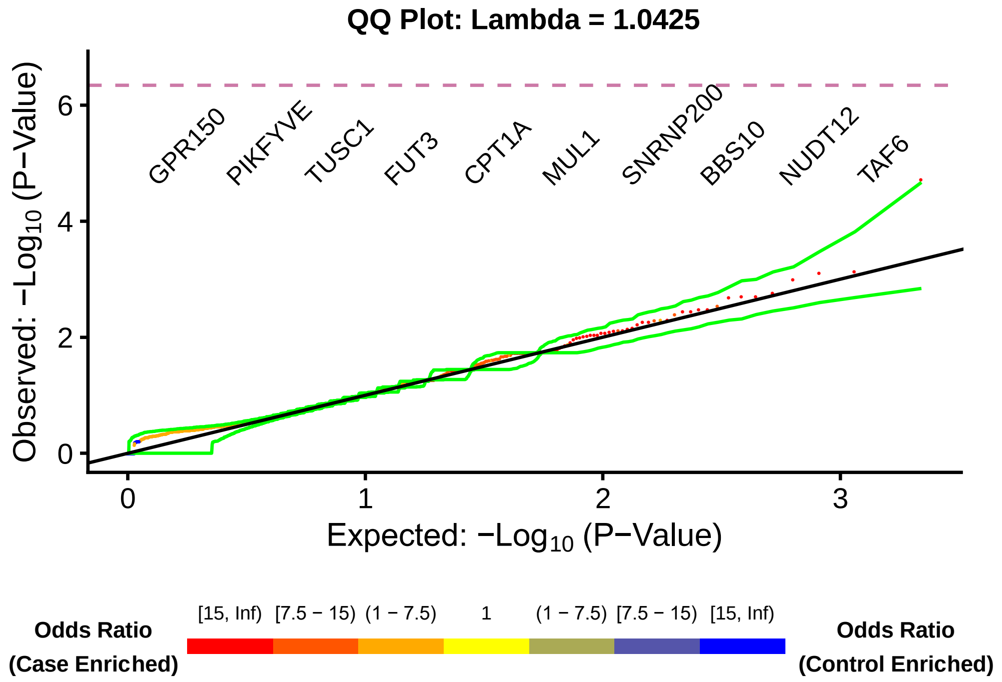

eFigure 2. *Ultra-Rare Synonymous Model Quantile-Quantile Plot for Combined Cohort*
The quantile-quantile plots for the protein-coding genes with at least one case or control carrier of an ultra-rare synonymous variant. All variants are ultra-rare (qualifying variants were defined as a minor allele frequency of less than 0.05% in internal case and control by cluster and absent in external reference cohorts). P-values were generated from the exact two-sided Cochran-Mantel-Haenszel (CMH) test by gene by cluster to indicate a different carrier status of cases in comparison to controls. Study-wide significance $p < 4.6 \times 10^{-7}$ after Bonferroni correction indicated by dashed line. Top ten case enriched genes are labeled. Point coloring determined by CMH odds ratio. The green lines represent the 95% confidence interval.

© 2022 Motelow JE et al. *JAMA Network Open.*

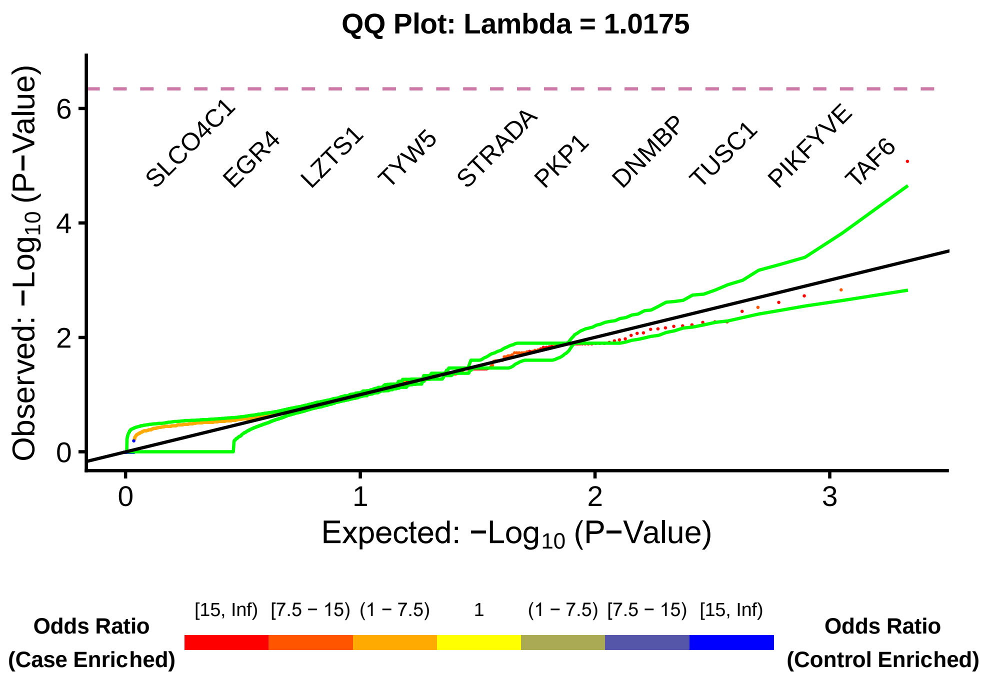

eFigure 3. *Ultra-Rare Synonymous Model Quantile-Quantile Plot for Unresolved Cohort*
The quantile-quantile plots for the protein-coding genes with at least one case or control carrier of an ultra-rare synonymous variant. All variants are ultra-rare (qualifying variants were defined as a minor allele frequency of less than 0.05% in internal case and control by cluster and absent in external reference cohorts). *P*-values were generated from the exact two-sided Cochran-Mantel-Haenszel (CMH) test by gene by cluster to indicate a different carrier status of cases in comparison to controls. Study-wide significance $p < 4.6 \times 10^{-7}$ after Bonferroni correction indicated by dashed line. Top ten case enriched genes are labeled. Point coloring determined by CMH odds ratio. The green lines represent the 95% confidence interval.

© 2022 Motelow JE et al. *JAMA Network Open.*

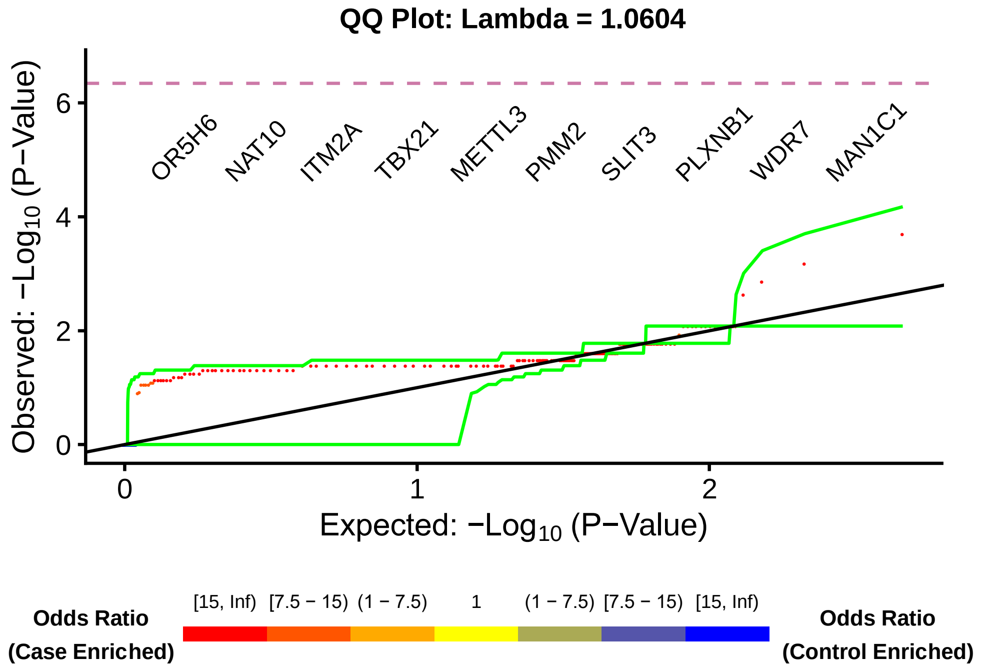

eFigure 4. *Ultra-Rare Synonymous Model Quantile-Quantile Plot for Respiratory Failure Cohort*
The quantile-quantile plots for the protein-coding genes with at least one case or control carrier of an ultra-rare synonymous variant. All variants are ultra-rare (qualifying variants were defined as a minor allele frequency of less than 0.05% in internal case and control by cluster and absent in external reference cohorts). P-values were generated from the exact two-sided Cochran-Mantel-Haenszel (CMH) test by gene by cluster to indicate a different carrier status of cases in comparison to controls. Study-wide significance $p < 4.6 \times 10^{-7}$ after Bonferroni correction indicated by dashed line. Top ten case enriched genes are labeled. Point coloring determined by CMH odds ratio. The green lines represent the 95% confidence interval.

© 2022 Motelow JE et al. *JAMA Network Open.*

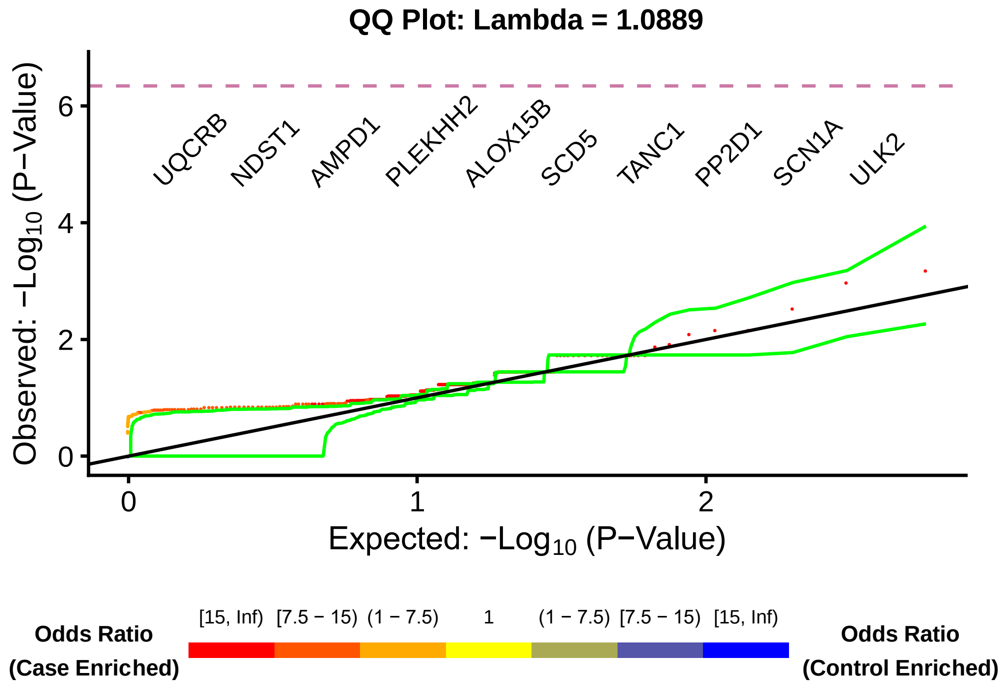

eFigure 5. *Ultra-Rare Loss-of-Function Model Quantile-Quantile Plot for Combined Cohort*
The quantile-quantile plots for the protein-coding genes with at least one case or control carrier of an ultra-rare loss-of-function variant. All variants are ultra-rare (qualifying variants were defined as a minor allele frequency of less than 0.05% in internal case and control by cluster and absent in external reference cohorts). P-values were generated from the exact two-sided Cochran-Mantel-Haenszel (CMH) test by gene by cluster to indicate a different carrier status of cases in comparison to controls. Study-wide significance $p < 4.6 \times 10^{-7}$ after Bonferroni correction indicated by dashed line. Top ten case enriched genes are labeled. Point coloring determined by CMH odds ratio. The green lines represent the 95% confidence interval.

© 2022 Motelow JE et al. *JAMA Network Open.*

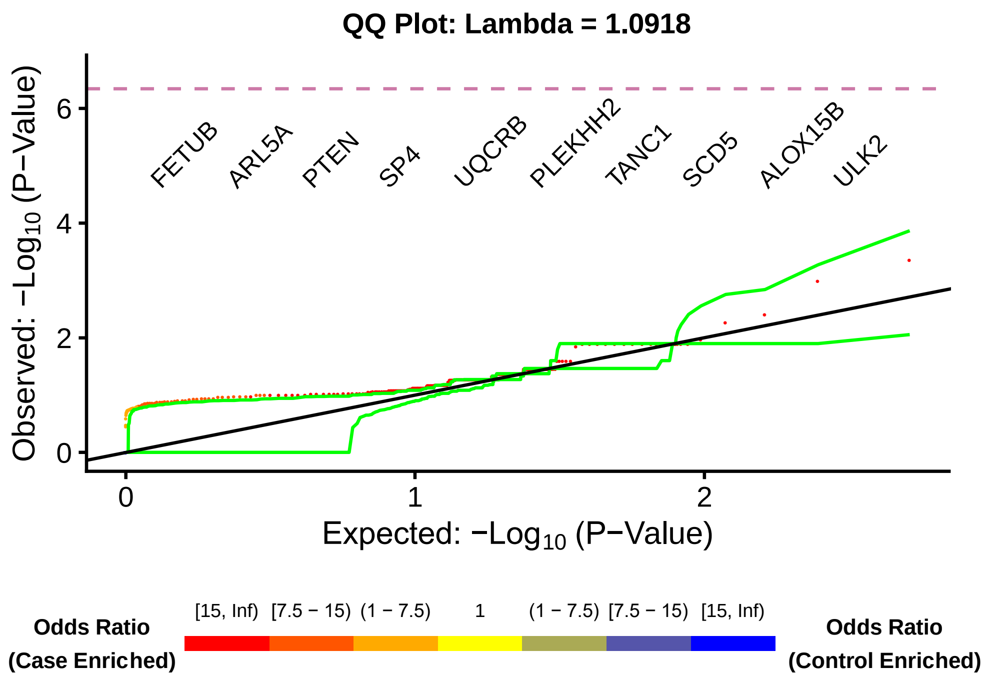

eFigure 6. *Ultra-Rare Loss-of-Function Model Quantile-Quantile Plot for Unresolved Cohort*
The quantile-quantile plots for the protein-coding genes with at least one case or control carrier of an ultra-rare loss-of-function variant. All variants are ultra-rare (qualifying variants were defined as a minor allele frequency of less than 0.05% in internal case and control by cluster and absent in external reference cohorts). P-values were generated from the exact two-sided Cochran-Mantel-Haenszel (CMH) test by gene by cluster to indicate a different carrier status of cases in comparison to controls. Study-wide significance $p < 4.6 \times 10^{-7}$ after Bonferroni correction indicated by dashed line (see Collapsing by Gene and Statistical Enrichment. Top ten case enriched genes are labeled. Point coloring determined by CMH odds ratio. The green lines represent the 95% confidence interval.

© 2022 Motelow JE et al. *JAMA Network Open.*

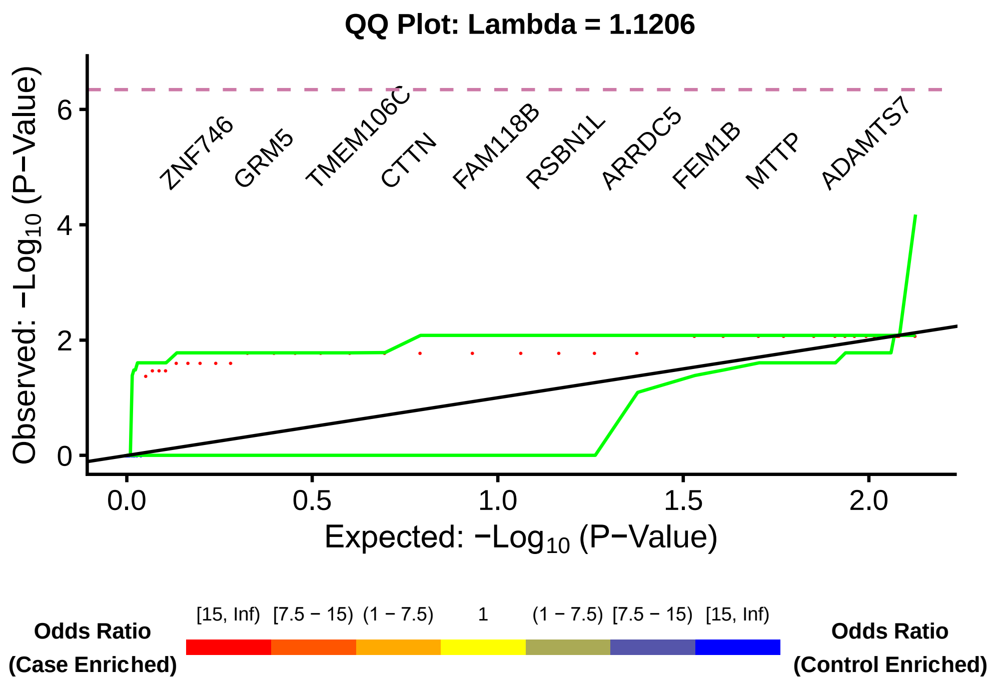

eFigure 7. *Ultra-Rare Loss-of-Function Model Quantile-Quantile Plot for Respiratory Failure*
The quantile-quantile plots for the protein-coding genes with at least one case or control carrier of an ultra-rare loss-of-function variant. All variants are ultra-rare (qualifying variants were defined as a minor allele frequency of less than 0.05% in internal case and control by cluster and absent in external reference cohorts). P-values were generated from the exact two-sided Cochran-Mantel-Haenszel (CMH) test by gene by cluster to indicate a different carrier status of cases in comparison to controls. Study-wide significance $p < 4.6 \times 10^{-7}$ after Bonferroni correction indicated by dashed line. Top ten case enriched genes are labeled. Point coloring determined by CMH odds ratio. The green lines represent the 95% confidence interval.

© 2022 Motelow JE et al. *JAMA Network Open.*

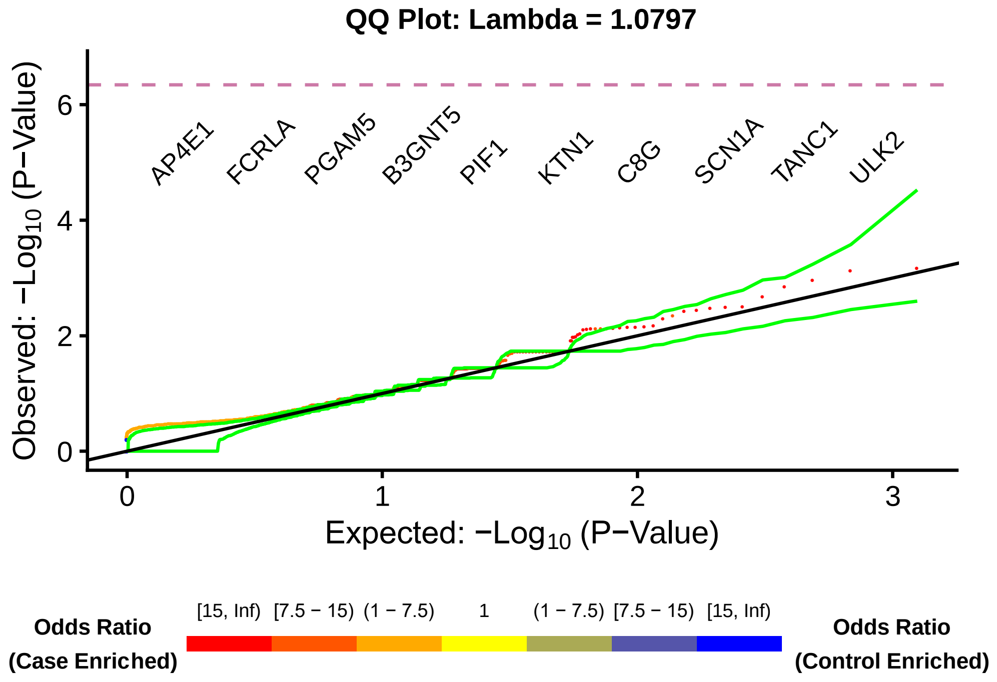

eFigure 8. *Flex Loss-of-Function Model Quantile-Quantile Plot for Combined*
The quantile-quantile plots for the protein-coding genes with at least one case or control carrier of a loss-of-function variant. All variants are ultra-rare (qualifying variants were defined as a minor allele frequency of less than 0.1% in external reference cohorts). P-values were generated from the exact two-sided Cochran-Mantel-Haenszel (CMH) test by gene by cluster to indicate a different carrier status of cases in comparison to controls. Study-wide significance $p < 4.6 \times 10^{-7}$ after Bonferroni correction indicated by dashed line. Top ten case enriched genes are labeled. Point coloring determined by CMH odds ratio. The green lines represent the 95% confidence interval.

© 2022 Motelow JE et al. *JAMA Network Open.*

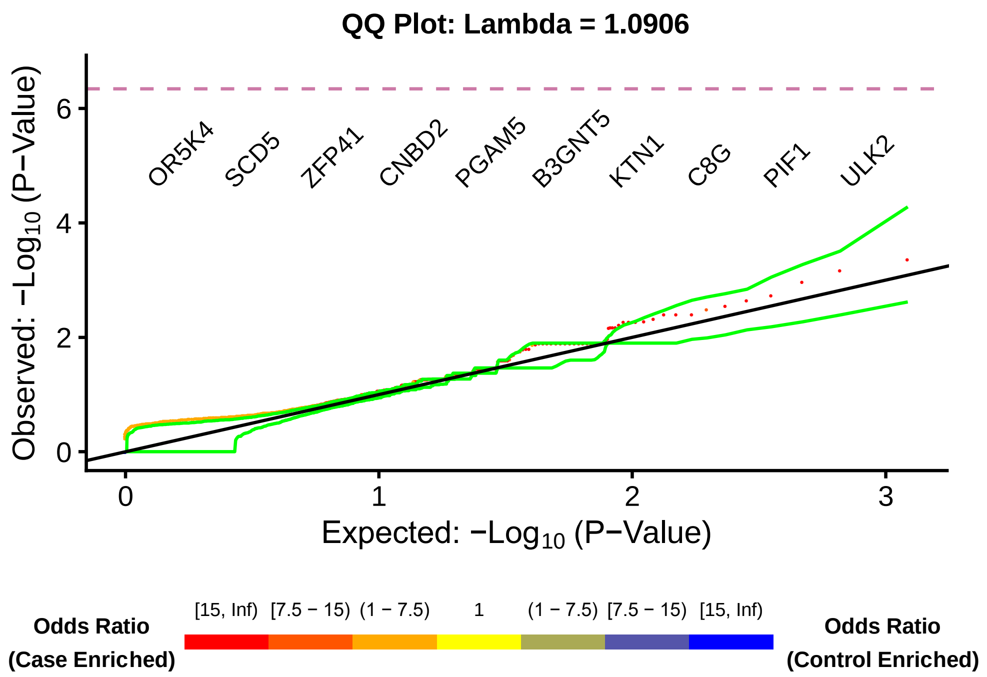

eFigure 9. *Flex Loss-of-Function Model Quantile-Quantile Plot for Unresolved Cohort*
The quantile-quantile plots for the protein-coding genes with at least one case or control carrier of a loss-of-function variant. All variants are ultra-rare (qualifying variants were defined as a minor allele frequency of less than 0.1% in external reference cohorts). P-values were generated from the exact two-sided Cochran-Mantel-Haenszel (CMH) test by gene by cluster to indicate a different carrier status of cases in comparison to controls. Study-wide significance $p < 4.6 \times 10^{-7}$ after Bonferroni correction indicated by dashed line. Top ten case enriched genes are labeled. Point coloring determined by CMH odds ratio. The green lines represent the 95% confidence interval.

© 2022 Motelow JE et al. *JAMA Network Open.*

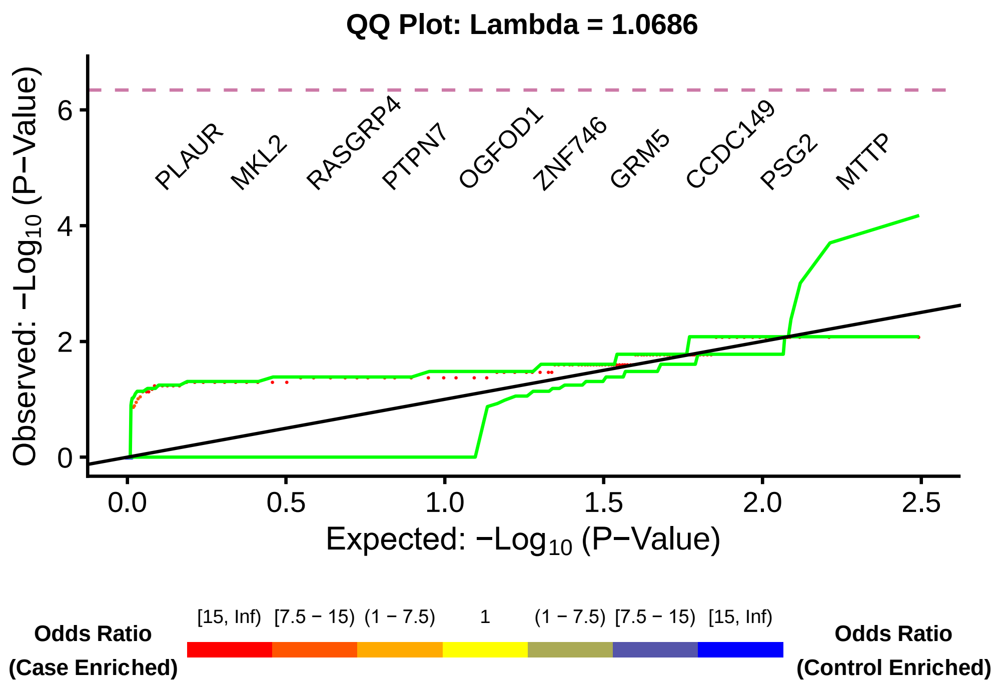

eFigure 10. *Flex Loss-of-Function Model Quantile-Quantile Plot for Respiratory Failure Cohort*
The quantile-quantile plots for the protein-coding genes with at least one case or control carrier of a loss-of-function variant. All variants are ultra-rare (qualifying variants were defined as a minor allele frequency of less than 0.1% in external reference cohorts). P-values were generated from the exact two-sided Cochran-Mantel-Haenszel (CMH) test by gene by cluster to indicate a different carrier status of cases in comparison to controls. Study-wide significance $p < 4.6 \times 10^{-7}$ after Bonferroni correction indicated by dashed line. Top ten case enriched genes are labeled. Point coloring determined by CMH odds ratio. The green lines represent the 95% confidence interval.

© 2022 Motelow JE et al. *JAMA Network Open.*

eMethods. Supplemental Methods

Definition of Critical Illness

All probands were critically ill as defined by meeting any of the following criteria: (1) admission to the pediatric intensive care unit (PICU) at Morgan Stanley Children's Hospital of NewYork-Presbyterian (MSCH) – Columbia University Irving Medical Center (CUIMC) (n = 264), (2) PICU admission at another institution per the medical record (n = 1), (3) death (n = 18), or (4) respiratory failure on the general pediatric floor requiring continuous positive airway pressure (n = 2). Included probands from MSCH/CUIMC) were enrolled either during admission to the pediatric intensive care unit (PICU) or a subsequent visit. In addition, three children were recruited from the general pediatric floor.

Respiratory Failure Cohort

The "respiratory failure cohort" was composed of probands from MSCH and OCME. MSCH probands were included if they had no contributing medical history of other than asthma or reactive airway disease which are difficult to diagnose in children less than 5,[2] had gestational age greater than or equal to 36 weeks (one proband did not have a gestational age included in the chart), had a common viral illness noted in the medical chart (eTable 2), and required invasive or non-invasive positive pressure ventilation (eTable 2). OCME probands were included if the limited medical history excluded any indication of chronic disease (eTable 1).

Controls

The same control samples were used as a base of comparison for each of the three case cohorts.

Phenotyping Children with Critical Illness

To qualitatively describe the heterogenous phenotypes present in the combined cohort, we mapped PICU admission notes (or general pediatric admission notes when no PICU note was available) to the Human Phenotype Ontology (HPO) (eTable 6).[3,4] We used doc2hpo for an initial conversion to HPO concept IDs and then further manually reviewed and edited the mapped concepts.[5] For the OCME cohort, limited clinical information was used (eTable 1).

WES and WGS Data Generation

Exomes were captured with multiple capture kits and sequenced according to standard protocols on Illumina's HiSeq 2000, HiSeq 2500, and NovaSeq 6000 (Illumina, San Diego, CA, USA) platform with 150 bp paired-end reads. Genomes were sequenced according to standard protocols on Illumina's NovaSeq 6000 (Illumina, San Diego, CA, USA) platform. Possible bias introduced by

© 2022 Motelow JE et al. *JAMA Network Open.*

using WES from different capture kits (eTable 22) and WGS in collapsing is corrected during coverage harmonization (see Collapsing Coverage Harmonization).[6,7]

Diagnostic Analysis

For diagnostic analysis, sample preparation, alignment and variant calling followed the same pipeline as in collapsing analysis (see WES and WGS Data Generation and Alignment and Variant Calling). WES data were analyzed using the qualifying genotype approach as previously been described previously. A combination of the American College of Medical Genetics and Association for Molecular Pathology variant classification as well as clinical context and clinical correlation from phenotype experts/referring providers was used to assess variant causality.[8-12] The OCME cohort was reviewed as well although limited clinical data made a full diagnostic analysis impossible. Probands were deemed resolve if their phenotype was fully or partially explained by either the research exome or any other clinical genetics data available by chart review. The summary of these data can be found in eTable 5.

Alignment and Variant Calling

Both affected individuals and control individuals were processed with the same IGM bioinformatic pipeline for variant calling. Reads were aligned to human reference GRCh37 via DRAGEN (Edico Genome, San Diego, CA, USA)[13] and duplicates were marked with Picard (Broad Institute, Boston, MA, USA). Variants were called according to the Genome Analysis Toolkit (GATK - Broad Institute, Boston, MA, USA) Best Practices recommendations v.3.6.[14,15] Finally, variants were annotated with ClinEff.[16] Custom annotations including Genome Aggregation Database (gnomAD) v.2.1 frequencies[17], Exome Aggregation Consortium (ExAC)[18] frequencies, and loss-of-function observed/expected upper bound fraction (LOEUF)[17] scores and deciles were added via the IGM's in-house analysis tool for annotated variants (ATAV) platform.[19] In the collapsing workflow, we relied on gene symbol matching between consensus coding sequence (CCDS release 20)[20] and our variant calling pipeline annotated by ClinEff. 18,286 CCDS v20 genes were called as part of the collapsing pipeline. 548 genes known to be part of the CCDS v20 were not called by the collapsing pipeline.

Collapsing Analysis Overview

The collapsing analysis implemented in this manuscript[6] takes the following steps: i) high-quality samples and selected controls with non-overlapping phenotypes are chosen and related individuals are removed (see Analyzed Cohort Definitions, Control Samples, and Collapsing Sample Quality Control), ii) match cases and controls in clusters based on geographic ancestry (see Collapsing Geographic Ancestry Clustering), iii) perform coverage harmonization separately in each cluster (see Collapsing Coverage Harmonization), iv) create models with specific QV criteria (see Table S3, Collapsing Variant Quality Control and Collapsing Model Specification), v) collapse by gene or gene set and assign indicator variable (0/1) to each case and control based on the absence/present of a QV in the gene or gene set, and vi) test for association between case/control status and indicator variable (see

Collapsing by Gene and Statistical Enrichment and Collapsing Gene-set Enrichment Testing), and vi) visualize results (see Collapsing Quantile-Quantile (QQ) Plots and Genomic Inflation Factor).

Collapsing Sample Quality Control

The same quality control standards were applied to cases and controls. We included only samples with at least 90% of the consensus coding sequence (CCDS release 20)[20] covered at a minimum of 10x, less or equal 2% contamination levels according to VerifyBamID[21], and single nucleotide variants (SNVs) and indels overlapping the Single Nucleotide Polymorphism database (dbSNP)[22] at least 85% and 80%, respectively. We excluded with a discordance between self-declared and sequence-derived gender to prevent phenotype-genotype mismatch. We utilized KING to detect related individuals and removed one of each pair that had an inferred relationship of second-degree or closer while favoring the inclusion of cases over controls and well-covered over poorly-covered.[23] Of the 285 samples in the combined cohort, three samples from the MSCH cohort were excluded due to low quality. No OCME samples were excluded.

Collapsing Geographic Ancestry Clustering

It is important to correct for the underlying rate of variation in samples of different geographic ancestry in case/control experimental designs.[24-26] The following steps were performed separately for each of the three analyzed case-control cohorts (see Analyzed Cohort Definitions). We performed principal component analysis on a set of pre-defined variants to capture population substructure. This was described by Cameron-Christie and colleagues.[27] We then utilized the Louvain method of community detection on the first six principal components to identify clusters reflecting the ancestry captured by the pre-defined variants mentioned above.[28,29] In parallel, a pre-trained neural-network generated probability estimates for each of six groups (European, African, Latino, East Asian, South Asian and Middle Eastern). A geographic ancestry label was assigned to each sample using a 95% probability cut-off. “Admixed” samples were those that did not reach 95% for any of the ancestry groups (eFigure 1).

To check the quality of the clusters, we performed further dimensionality reduction using the Uniform Manifold Approximation and Projection (UMAP)[30] on the first six PCs to disentangle subcontinental structure, which is then reflected in the cluster membership.[31,32] In order to assess the quality of clustering, we used UMAP to visualize overlap between cluster membership and predicted geographic ancestry. UMAP was not itself used for clustering. One can see in eFigure 1 that, given adequate sample size, the Louvain method creates clusters that are consistent with predicted ancestry. This provides evidence that the clusters produced by the Louvain method are adhering to geographic ancestry.

Clusters containing at least 20 cases and 20 controls were used for all analyses based on collapsing clusters (Figures 1, 2, and 3) [7] All clusters underwent coverage harmonization (see Collapsing coverage harmonization in Methods).

The combined and unresolved cohorts included multiple clusters and used the Cochran-Mantel-Haenszel test for statistical association testing. The respiratory failure cohort included only one cluster and used Fisher's exact test for statistical association testing.

© 2022 Motelow JE et al. *JAMA Network Open.*

Collapsing Coverage Harmonization

To ensure that the same genomic sequences are considered in both cases and controls, our collapsing pipeline uses "coverage harmonization".[6] Coverage differences between cases and controls introduce bias because variants can only be called with sufficient coverage. It is important to ensure that all statistical tests measuring association between case/control status and qualifying variants examine the same areas of the genome in both cases and controls. If areas of the genome are considered in cases but not controls, a spurious association may be made. Our approach to control for this bias is the following. First, we ensure that only samples with adequate coverage are considered. We include only cases and controls for which 90% of the consensus coding sequence (CCDS release 20) is covered at more than 10x. This ensures the inclusion of only well-covered samples. Second, we evaluate protein coding sites only. Third, we considered only genomic sites that are covered more than 10x. Fourth, we used a site-based pruning approach and removed sites where the absolute difference in percentages of cases compared to controls with at least 10x coverage was greater than 7.0%. Without "coverage harmonization", it is possible that a variant can be susceptible to false-negative calls in the less well-represented group. This would lead to bias and enrichment towards the more represented group. This process reduces the influence of coverage differences caused by different capture kits, inclusion of both WES and WGS or sequencing depth in general. This methodology has been used in prior publications from our lab.[7,33,34] We performed coverage harmonization on each cluster independently (see Collapsing Geographic Ancestry Clustering). This resulted in three sets coverage maps (eFigure 1).

Collapsing Variant Quality Control

In each cluster, we called variants at bases available for variant calling per cluster-specific coverage harmonization (see Collapsing Coverage Harmonization). Only variants meeting the following qualifications were considered for analysis: i) at least 10x coverage of the site, ii) quality score (QUAL) ≥ 50, iii) genotype quality score (GQ) ≥ 20, iv) quality by depth score (QD) ≥ 5, v) mapping quality score (MQ) ≥ 40, vi) read position rank sum score (RPRS) ≥ -3, vii) mapping quality rank sum score (MQRS) ≥ -10, viii) Fisher's strand bias score (FS) ≤ 60 (SNVs) or ≤ 200 (indels), ix) strand odds ratio (SOR) ≤ 3 (SNVs) or ≤ 10 (indels), x) GATK Variant Quality Score Recalibration filter "PASS", xi) alternate allele fraction for heterozygous calls ≥ 0.3, xii) within the CCDS inclusive of two base intronic extensions to accommodate canonical splice variants, xiii) a proportion expression across transcripts (pext) value (when available) greater than or equal to 1/10 the maximum pext value for that gene were removed as they are unlikely to affect translated mRNA[35], and xiv) located outside regions with highly repetitive elements to reduce false-positivity.[36] Sequencing artifacts as described previously[37] and low quality variants per Exome Aggregation Consortium[38], gnomAD[17], or the Exome Variant Server were excluded (see Web Resources). All predicted loss-of-function (LOF) variants (stop gain, frameshift, splice acceptor, and splice donor variants) were filtered with Loss-Of-Function Transcript Effect Estimator (LOFTEE) to remove likely false-positive LOFs.[17]

Collapsing Model Specification

Each collapsing model depends on the definition of a qualifying variant (QV) with parameters designed to enriched for real variant calls with strong functional effects (eTable 3).[6,39] Model parameters included external minor allele frequency (MAF), internal allele

© 2022 Motelow JE et al. *JAMA Network Open.*

frequency, variant effect, and *in-silico filters*. External frequency filters in gnomAD and ExAC which could be either “ultra-rare” (absent) or “flex” (MAF < 0.1%). For the flex model, MAF was filtered at a population specific level. For ExAC, populations included afr, amr, nfe, fin, eas, sas. For gnomAD exomes, populations included afr, amr, asj, eas, sas, fin, and nfe. For gnomAD genomes, the MAF filter was applied to the full population. For the purposes of gene-set analysis (Figures 2 and 3), a further subset “rare but public” was defined which removed all ultra-rare variants leaving only variants that are rare but still present in gnomAD or ExAC. Internal allele frequencies were applied by cluster. For ultra-rare models, variants were excluded with an internal allele frequency greater than 0.05% applied to the combined case-control call set by cluster excluding one allele to allow for clusters in which one allele might exceed that allele frequency threshold.[35] For flex models, the internal allele frequency filter was set at 0.1%.

Collapsing by Gene and Statistical Enrichment

From the collapsing matrices of each cluster, we extracted the number of cases/controls with and without a QV per gene and used the exact two-sided Cochran-Mantel-Haenszel (CMH) test to test for an enrichment of qualifying variants in the case or control group (eTable 2) while controlling for cluster membership.[7,29,40,41] (2) The respiratory failure cohort included only one cluster large enough for analysis. For models analyzed for this cohort, an individual-by-gene matrix was created as above for the single cluster. We implemented a two-tailed Fisher’s exact test to identify genes where there was a significant enrichment of qualifying variants in the case or control group.[42]

To visualize our results and ensure appropriate genomic inflation, we created quantile-quantile (QQ) plots (described below). The synonymous model was used as a putatively negative control for each cohort (eFigures 2 - 4, eTables 8 – 10). We defined a study-wide Bonferroni multiplicity-adjusted significance threshold of $p < 4.6 \times 10^{-7}$ (0.05 / [18286 CCDS genes × 6 non-synonymous models]). Model details for the six non-synonymous models can be found in eTable 3. The top 10 ranked genes for all nine models can be found in the supplemental tables (eTables 8 - 16). The membership of each gene in the following gene-sets is also indicated: (O) disease association (see Gene-Set Enrichment Testing).

Collapsing Quantile-Quantile (QQ) Plots and Genomic Inflation Factor λ

For each model, we plotted expected vs. observed *p*-values for our collapsing by gene enrichment results. We generated empirical (permutation-based) expected probability distributions using one of two methods for each model independently. (1) For models using the combined and unresolved cohort which used multiple clusters, we used a process previously described.[7,29] For each cluster, the original case and control labels were randomly permuted while the rest of the gene by sample matrix was kept fixed. For each cluster we extracted the number of newly labeled cases/controls with and without a QV per gene and used the CMH test to test for an association between case/control status and QV status while controlling for cluster membership (see Collapsing by Gene and Statistical Enrichment). This process was repeated 1,000 times to create an empirical distribution of 1,000 *p*-values for each gene, and for each permutation the *p*-values were ordered. (2) For the respiratory failure cohort in which only one cluster was used, we randomly permutated the case/control labels in the single cluster.[6,34,37,43] After each permutation, A two-tailed Fisher’s exact test was performed to test for an association between case/control status and QV status (see Collapsing by gene and statistical enrichment).

© 2022 Motelow JE et al. *JAMA Network Open.*

This process was repeated 1,000 times to create an empirical distribution of 1,000 *p*-values for each gene and for each permutation, the *p*-values were ordered.

Empirical estimates of the expected ordered *p*-values were represented by the mean of each rank-ordered estimate across the 1,000 permutations (i.e., the average 1st order statistic, the average 2nd order statistic, etc.). The negative logarithms of the expected and observed *p*-values were plotted to get permutation-based QQ plots. We estimated the genomic inflation factor λ based on the permutation-based expected *p*-values using a regression method as described previously.[37,42]

Collapsing Gene-Set Enrichment Testing

Biologically informed gene-sets can reveal important pathways or gene characteristics by aggregated signal across related genes.[6,42] Association between case/control status and harboring a variant in a gene-set was tested in two ways. (1) For the combined and unresolved cohorts which included multiple clusters, we extracted the number of cases/controls with and without at least one QV among any of the genes in each of the gene-sets and used the exact two-sided CMH test[7,40,41] to test for an association between case/control status and QV status while controlling for cluster membership. (2) For the respiratory failure cohort which included only one cluster, we extracted the number of cases/controls with and without at least one QV among any of the genes in each of the gene-sets and used a two-tailed Fisher's exact test to test for an association between case/control status and QV status.[42]

We used a false discovery rate (FDR) adjustment for multiple comparisons. We performed 22 CMH tests or FETs to determine odds ratios for gene-set enrichment testing (eTables 17 - 20) and defined a significant enrichment at FDR < 0.05.

Unbiased LOF Enrichment Analysis

Given the rarity of pediatric critical illness, we hypothesized that the combined cohort would be more likely to harbor LOF variants than controls and that these variants would exist in genes "intolerant" to variation (i.e., genes in which few LOF variants are found in otherwise healthy individuals).[17,44] The empirical *p*-values of the 1,860 tests were determined by permutation. We randomly shuffled the case/control labels within each cluster and re-calculated the *p*-value. This was performed 100,000 times leaving 100,000 permuted *p*-values for each of the 1,860 gene-sets. The empirically derived *p*-value at each gene-set was then determined by the fraction of permuted *p*-values less than the actual *p*-value for that gene-set. We identified the LOEUF value with the most significant threshold and used the gene-set defined by this LOEUF threshold for forest plots (see Collapsing Gene-Set Enrichment Testing).

De Novo Mutation Calling, Filtering and Analysis

We used ATAV's "—list-trio" function (ATAV v7.2.1) to screen for *de novo* variants.
The initial set of candidate *de novo* variants met the following quality control thresholds: i) AD Alt ≥3, ii) QC Filter Pass, iii) QUAL ≥ 50, iv) GQ ≥ 20, v) MQ ≥ 40, vi) variant site covered in both parents with at least 10 reads, vii) variant absent in parents, viii) variant absent in gnomAD exome and genome controls, ix) absent in IGM controls, and x) child het carrier ≥ 10% alt read OR child hom carrier ≥ 80% alt read. To further improve the quality of *de novo* calls, we imposed additional criteria: i) variant located outside

© 2022 Motelow JE et al. *JAMA Network Open.*

regions with highly repetitive elements to reduce false-positivity,[36] and ii) SNV VQSR tranche < 99. Finally, all loss-of-function *de novo* variants were visually inspected in IGV to confirm the underlying alignment. Any variant call that failed visual inspection was excluded.

Using denovolyzeR, synonymous, missense and loss-of-function are analyzed in addition to combining missense and loss-of-function to capture protein-altering variants.[45-47], The tool allows for analyses of all genes or gene-sets. We focused on genes without a disease association. Loss-of-function variants were those with frameshift, splice_acceptor_variant, splice_donor_variant, and stop_gained effects. First, we updated the table of per-gene mutation rates to those used in DeNovoWEST published in 2020.[48] This is the same group which developed *denovolyzeR*, and the mutation rates per gene reflect developments since the original development of *denovolyzeR* in 2014.[45] The updated mutation rates were derived from data on https://github.com/HurlesGroupSanger/DeNovoWEST.

Determination of Disease-Gene Association

Gene-disease associations were determined from the three databases. (1) Online Mendelian Inheritance in Man (OMIM, see web resources).[49] genemap2.txt was downloaded on 8/25/2022. Genes were filtered to remove those with blank phenotypes or phenotypes that begin with "?" (indicating a relationship between the phenotype and gene is provisional) or "[" (indicating a non-disease phenotype). The remaining genes are considered to be associated with disease. Using this method, 4,145 genes were determined to have a disease association in OMIM and exist in our variant calling pipeline. (2) We downloaded the developmental disorder (DD) gene list from the Development Disorder Genotype - Phenotype Database (DDG2P) website (https://www.ebi.ac.uk/gene2phenotype/downloads) on 8/28/2022.[50] We included genes with "Strong" or "Definitive" evidence (877 genes). (3) We downloaded an autism spectrum disorder (ASD) gene list from The Human Gene module of SFARI Gene (https://gene.sfari.org/database/human-gene/).[51,52] We included genes with either High Confidence (category 1) or Strong Candidate (category 2) evidence (1,898 genes). There was overlap between these two additional gene lists and the OMIM gene list (408 from the ASD and 1,836 from the DD gene lists). Ultimately, our gene list for known disease associations included 4,662 unique genes. The remaining genes were deemed to be without a disease association.

The "Primary Immunodeficiency" gene list was 424 genes drawn from the Invitae Primary Immunodeficiency Panel (Test code: 08100, https://www.invitae.com/en/providers/test-catalog/test-08100). The Viral Immunodeficiency gene list was 13 genes drawn from Zhang et al.[1] The primary ciliary dyskinesia gene list was drawn from the GeneDx Primary Ciliary Dyskinesia Panel (https://www.genedx.com/tests/detail/primary-ciliary-dyskinesia-1073). The asthma gene list was a combination of OMIM genes with "asthma" in the phenotype (17 genes), asthma gene set provided by Kyoto Encyclopedia of Genes and Genomes (KEGG, https://www.genome.jp/kegg/pathway/hsa/hsa05310.html) (30 genes), and all genes associated with asthma in the National Institute of Health Genetic Testing Registry on 9/5/2022 (https://www.ncbi.nlm.nih.gov/gtr/) (81 genes).[53] Combined, these lists included 101 unique genes in our collapsing pipeline.

Gene lists are available at https://github.com/moe1619/picu_wes_unresolved

Data Analysis and Display

Unless otherwise noted in the methods, data analysis and visualization were performed with R (v.3.6.0).[54]

© 2022 Motelow JE et al. *JAMA Network Open.*

eReferences

1. Zhang Q, Bastard P, Liu Z, et al. Inborn errors of type I IFN immunity in patients with life-threatening COVID-19. *Science.* 2020;370(6515).
2. Martinez FD, Wright AL, Taussig LM, Holberg CJ, Halonen M, Morgan WJ. Asthma and wheezing in the first six years of life. The Group Health Medical Associates. *N Engl J Med.* 1995;332(3):133-138.
3. Kohler S, Gargano M, Matentzoglu N, et al. The Human Phenotype Ontology in 2021. *Nucleic Acids Res.* 2021;49(D1):D1207-D1217.
4. Robinson PN, Kohler S, Bauer S, Seelow D, Horn D, Mundlos S. The Human Phenotype Ontology: a tool for annotating and analyzing human hereditary disease. *Am J Hum Genet.* 2008;83(5):610-615.
5. Liu C, Peres Kury FS, Li Z, Ta C, Wang K, Weng C. Doc2Hpo: a web application for efficient and accurate HPO concept curation. *Nucleic Acids Res.* 2019;47(W1):W566-W570.
6. Povysil G, Petrovski S, Hostyk J, Aggarwal V, Allen AS, Goldstein DB. Rare-variant collapsing analyses for complex traits: guidelines and applications. *Nat Rev Genet.* 2019;20(12):747-759.
7. Epi25 Collaborative. Electronic address jcce, Epi C. Sub-genic intolerance, ClinVar, and the epilepsies: A whole-exome sequencing study of 29,165 individuals. In. *Am J Hum Genet*. 2021/05/02 ed2021.
8. Need AC, Shashi V, Hitomi Y, et al. Clinical application of exome sequencing in undiagnosed genetic conditions. *J Med Genet.* 2012;49(6):353-361.
9. Zhu X, Petrovski S, Xie P, et al. Whole-exome sequencing in undiagnosed genetic diseases: interpreting 119 trios. *Genet Med.* 2015;17(10):774-781.
10. Petrovski S, Aggarwal V, Giordano JL, et al. Whole-exome sequencing in the evaluation of fetal structural anomalies: a prospective cohort study. *Lancet.* 2019;393(10173):758-767.
11. Lippa N, Bier L, Revah-Politi A, et al. Diagnostic sequencing to support genetically stratified medicine in a tertiary care setting. *Genet Med.* 2022.
12. Richards S, Aziz N, Bale S, et al. Standards and guidelines for the interpretation of sequence variants: a joint consensus recommendation of the American College of Medical Genetics and Genomics and the Association for Molecular Pathology. *Genet Med.* 2015;17(5):405-424.
13. Miller NA, Farrow EG, Gibson M, et al. A 26-hour system of highly sensitive whole genome sequencing for emergency management of genetic diseases. *Genome Med.* 2015;7:100.
14. McKenna A, Hanna M, Banks E, et al. The Genome Analysis Toolkit: a MapReduce framework for analyzing next-generation DNA sequencing data. *Genome Res.* 2010;20(9):1297-1303.
15. Van der Auwera GA, Carneiro MO, Hartl C, et al. From FastQ data to high confidence variant calls: the Genome Analysis Toolkit best practices pipeline. *Curr Protoc Bioinformatics.* 2013;43(1):11 10 11-11 10 33.
16. Cingolani P, Platts A, Wang le L, et al. A program for annotating and predicting the effects of single nucleotide polymorphisms, SnpEff: SNPs in the genome of Drosophila melanogaster strain w1118; iso-2; iso-3. *Fly (Austin).* 2012;6(2):80-92.
17. Karczewski KJ, Francioli LC, Tiao G, et al. The mutational constraint spectrum quantified from variation in 141,456 humans. *Nature.* 2020;581(7809):434-443.

© 2022 Motelow JE et al. *JAMA Network Open.*

18. Lek M, Karczewski KJ, Minikel EV, et al. Analysis of protein-coding genetic variation in 60,706 humans. *Nature.* 2016;536(7616):285-291.
19. Ren Z, Povysil G, Hostyk JA, Cui H, Bhardwaj N, Goldstein DB. ATAV: a comprehensive platform for population-scale genomic analyses. *BMC Bioinformatics.* 2021;22(1):149.
20. Pruitt KD, Harrow J, Harte RA, et al. The consensus coding sequence (CCDS) project: Identifying a common protein-coding gene set for the human and mouse genomes. *Genome Res.* 2009;19(7):1316-1323.
21. Jun G, Flickinger M, Hetrick KN, et al. Detecting and estimating contamination of human DNA samples in sequencing and array-based genotype data. *Am J Hum Genet.* 2012;91(5):839-848.
22. Sayers EW, Barrett T, Benson DA, et al. Database resources of the National Center for Biotechnology Information. *Nucleic Acids Res.* 2011;39(Database issue):D38-51.
23. Manichaikul A, Mychaleckyj JC, Rich SS, Daly K, Sale M, Chen WM. Robust relationship inference in genome-wide association studies. *Bioinformatics.* 2010;26(22):2867-2873.
24. Novembre J, Johnson T, Bryc K, et al. Genes mirror geography within Europe. *Nature.* 2008;456(7218):98-101.
25. Hellwege JN, Keaton JM, Giri A, Gao X, Velez Edwards DR, Edwards TL. Population Stratification in Genetic Association Studies. *Curr Protoc Hum Genet.* 2017;95:1 22 21-21 22 23.
26. Genomes Project C, Auton A, Brooks LD, et al. A global reference for human genetic variation. *Nature.* 2015;526(7571):68-74.
27. Cameron-Christie S, Wolock CJ, Groopman E, et al. Exome-Based Rare-Variant Analyses in CKD. *J Am Soc Nephrol.* 2019;30(6):1109-1122.
28. Blondel VD, Guillaume JL, Lambiotte R, Lefebvre E. Fast unfolding of communities in large networks. *Journal of Statistical Mechanics-Theory and Experiment.* 2008;2008(10):P10008.
29. Povysil G, Chazara O, Carss KJ, et al. Assessing the Role of Rare Genetic Variation in Patients With Heart Failure. *JAMA Cardiol.* 2021;6(4):379-386.
30. McInnes L, Healy J, Melville J. UMAP: Uniform Manifold Approximation and Projection for Dimension Reduction. *arXiv* https://arxivorg/abs/180203426. 2018.
31. Diaz-Papkovich A, Anderson-Trocme L, Ben-Eghan C, Gravel S. UMAP reveals cryptic population structure and phenotype heterogeneity in large genomic cohorts. *PLoS Genet.* 2019;15(11):e1008432.
32. Dai CL, Vazifeh MM, Yeang CH, et al. Population Histories of the United States Revealed through Fine-Scale Migration and Haplotype Analysis. *Am J Hum Genet.* 2020;106(3):371-388.
33. Zoghbi AW, Dhindsa RS, Goldberg TE, et al. High-impact rare genetic variants in severe schizophrenia. *Proc Natl Acad Sci U S A.* 2021;118(51).
34. Gelfman S, Dugger S, de Araujo Martins Moreno C, et al. A new approach for rare variation collapsing on functional protein domains implicates specific genic regions in ALS. *Genome Res.* 2019;29(5):809-818.
35. Cummings BB, Karczewski KJ, Kosmicki JA, et al. Transcript expression-aware annotation improves rare variant interpretation. *Nature.* 2020;581(7809):452-458.
36. Krusche P, Trigg L, Boutros PC, et al. Best practices for benchmarking germline small-variant calls in human genomes. *Nat Biotechnol.* 2019;37(5):555-560.
37. Petrovski S, Todd JL, Durheim MT, et al. An Exome Sequencing Study to Assess the Role of Rare Genetic Variation in Pulmonary Fibrosis. *Am J Respir Crit Care Med.* 2017;196(1):82-93.

© 2022 Motelow JE et al. *JAMA Network Open.*

38. Gravel S, Henn BM, Gutenkunst RN, et al. Demographic history and rare allele sharing among human populations. *Proc Natl Acad Sci U S A.* 2011;108(29):11983-11988.
39. Cirulli ET, Goldstein DB. Uncovering the roles of rare variants in common disease through whole-genome sequencing. *Nat Rev Genet.* 2010;11(6):415-425.
40. Mantel N, Haenszel W. Statistical aspects of the analysis of data from retrospective studies of disease. *J Natl Cancer Inst.* 1959;22(4):719-748.
41. Cochran WG. Some Methods for Strengthening the Common X2 Tests. *Biometrics.* 1954;10(4):417-451.
42. Epi Kc, Epilepsy Phenome/Genome P. Ultra-rare genetic variation in common epilepsies: a case-control sequencing study. In. *Lancet Neurol.* Vol 16. 2017/01/20 ed2017:135-143.
43. Stanley KE, Giordano J, Thorsten V, et al. Causal Genetic Variants in Stillbirth. *N Engl J Med.* 2020;383(12):1107-1116.
44. Petrovski S, Wang Q, Heinzen EL, Allen AS, Goldstein DB. Genic intolerance to functional variation and the interpretation of personal genomes. *PLoS Genet.* 2013;9(8):e1003709.
45. Ware JS, Samocha KE, Homsy J, Daly MJ. Interpreting de novo Variation in Human Disease Using denovolyzeR. *Curr Protoc Hum Genet.* 2015;87:7 25 21-27 25 15.
46. Halvorsen M, Gould L, Wang X, et al. De novo mutations in childhood cases of sudden unexplained death that disrupt intracellular Ca(2+) regulation. *Proc Natl Acad Sci U S A.* 2021;118(52).
47. Samocha KE, Robinson EB, Sanders SJ, et al. A framework for the interpretation of de novo mutation in human disease. *Nat Genet.* 2014;46(9):944-950.
48. Kaplanis J, Samocha KE, Wiel L, et al. Evidence for 28 genetic disorders discovered by combining healthcare and research data. *Nature.* 2020;586(7831):757-762.
49. Medicine M-NIoG. Online Mendelian Inheritance in Man, OMIM®. Johns Hopkins University. https://omim.org/. Published 2019. Accessed.
50. Thormann A, Halachev M, McLaren W, et al. Flexible and scalable diagnostic filtering of genomic variants using G2P with Ensembl VEP. *Nat Commun.* 2019;10(1):2373.
51. Banerjee-Basu S, Packer A. SFARI Gene: an evolving database for the autism research community. *Dis Model Mech.* 2010;3(3-4):133-135.
52. Abrahams BS, Arking DE, Campbell DB, et al. SFARI Gene 2.0: a community-driven knowledgebase for the autism spectrum disorders (ASDs). *Mol Autism.* 2013;4(1):36.
53. Rubinstein WS, Maglott DR, Lee JM, et al. The NIH genetic testing registry: a new, centralized database of genetic tests to enable access to comprehensive information and improve transparency. *Nucleic Acids Res.* 2013;41(Database issue):D925-935.
54. Team RC. *R: A Language and Environment for Statistical Computing.* Vienna, Austria: R Foundation for Statistical Computing; 2019.

© 2022 Motelow JE et al. *JAMA Network Open.*
